# Genomic characterization of *Pseudomonas* spp. on food: implications for spoilage, antimicrobial resistance and human infection

**DOI:** 10.1186/s12866-023-03153-9

**Published:** 2024-01-11

**Authors:** Samuel J. Bloomfield, Raphaёlle Palau, Emma R. Holden, Mark A. Webber, Alison E. Mather

**Affiliations:** 1 Quadram Institute Bioscience, Norwich Research Park, Norwich, UK; 2 University of East Anglia, Norwich, UK

**Keywords:** *Pseudomonas* spp., *Pseudomonas aeruginosa*, Food, Spoilage, Antimicrobial resistance, Whole genome sequencing

## Abstract

**Background:**

*Pseudomonas* species are common on food, but their contribution to the antimicrobial resistance gene (ARG) burden within food or as a source of clinical infection is unknown. *Pseudomonas aeruginosa* is an opportunistic pathogen responsible for a wide range of infections and is often hard to treat due to intrinsic and acquired ARGs commonly carried by this species. This study aimed to understand the potential role of *Pseudomonas* on food as a reservoir of ARGs and to assess the presence of potentially clinically significant *Pseudomonas aeruginosa* strains on food. To achieve this, we assessed the genetic relatedness (using whole genome sequencing) and virulence of food-derived isolates to those collected from humans.

**Results:**

A non-specific culturing approach for *Pseudomonas* recovered the bacterial genus from 28 of 32 (87.5%) retail food samples, although no *P. aeruginosa* was identified. The *Pseudomonas* species recovered were not clinically relevant, contained no ARGs and are likely associated with food spoilage. A specific culture method for *P. aeruginosa* resulted in the recovery of *P. aeruginosa* from 14 of 128 (11%) retail food samples; isolates contained between four and seven ARGs each and belonged to 16 sequence types (STs), four of which have been isolated from human infections. Food *P. aeruginosa* isolates from these STs demonstrated high similarity to human-derived isolates, differing by 41–312 single nucleotide polymorphisms (SNPs). There were diverse *P. aeruginosa* collected from the same food sample with distinct STs present on some samples and isolates belonging to the same ST differing by 19–67 SNPs. The *Galleria mellonella* infection model showed that 15 of 16 STs isolated from food displayed virulence between a low-virulence (PAO1) and a high virulence (PA14) control.

**Conclusion:**

The most frequent *Pseudomonas* recovered from food examined in this study carried no ARGs and are more likely to play a role in food spoilage rather than infection. *P. aeruginosa* isolates likely to be able to cause human infections and with multidrug resistant genotypes are present on a relatively small but still substantial proportions of retail foods examined. Given the frequency of exposure, the potential contribution of food to the burden of *P. aeruginosa* infections in humans should be evaluated more closely.

**Supplementary Information:**

The online version contains supplementary material available at 10.1186/s12866-023-03153-9.

## Background

The *Pseudomonas* genus includes important plant pathogens [1], agents of food spoilage [2] and opportunistic pathogens to animals and humans [3]. Previous metagenomic analyses have shown that *Pseudomonas* is the predominant bacterial genus found on retail food, including seafood and foods of plant and animal origin [4]. Bacteria on food may also carry antimicrobial resistance genes (ARGs), which may be transferred to other bacteria on food [5]. To date, few studies have described the genetic diversity of *Pseudomonas* on food and how this contributes to the clinical burden of disease either as a pathogen or as a potential reservoir of ARGs [6].

A total of 313 *Pseudomonas* species have been described and published [7], but the most clinically significant species is *Pseudomonas aeruginosa*, an opportunistic pathogen of humans, commonly associated with nosocomial infections [8], burn wound infections [9] and pneumonia in cystic fibrosis patients [10]. *P. aeruginosa* infections are difficult to treat as the species contains multiple intrinsic antimicrobial resistance (AMR) mechanisms, and readily adapts to antimicrobial pressure by accumulating mutations or acquiring ARGs via horizontal gene transfer [11]. Sources of *P. aeruginosa* infections are hard to identify due to the ubiquitous nature of the bacterium [12], which is found in soil and water environments, particularly those associated with human activity [13].

*P. aeruginosa* has also been isolated from diverse food types [14, 15] and *P. aeruginosa* from foods in hospitals has been identified as a potential source of nosocomial infections [16]. *P. aeruginosa* has been shown to translocate from the gastrointestinal tract to the lung [17], which could precede these infections.

Whole genome sequencing (WGS) is the most discriminatory method available to distinguish bacteria, and can be used to identify and characterize the genetic content of isolates, and assess putative sources of infections [18]. The aim of this study was to understand the potential role of *Pseudomonas* on food as a reservoir of ARGs for foodborne pathogens and to determine the prevalence of potentially clinically significant *P. aeruginosa* strains on food.

## Results

### *Pseudomonas* spp. analysis

We initially attempted to isolate *P. aeruginosa* from food by non-specifically culturing for *Pseudomonas* spp. *Pseudomonas* spp. presence was tested in 32 food samples and was isolated from all leafy green (8/8) and prawn samples (4/4), 7/8 of chicken, 7/8 pork samples, and 2/4 salmon samples. A total of 93 *Pseudomonas* isolates were cultured and sequenced from the 28 positive samples (Supplementary Table 1).

**Fig. 1:**
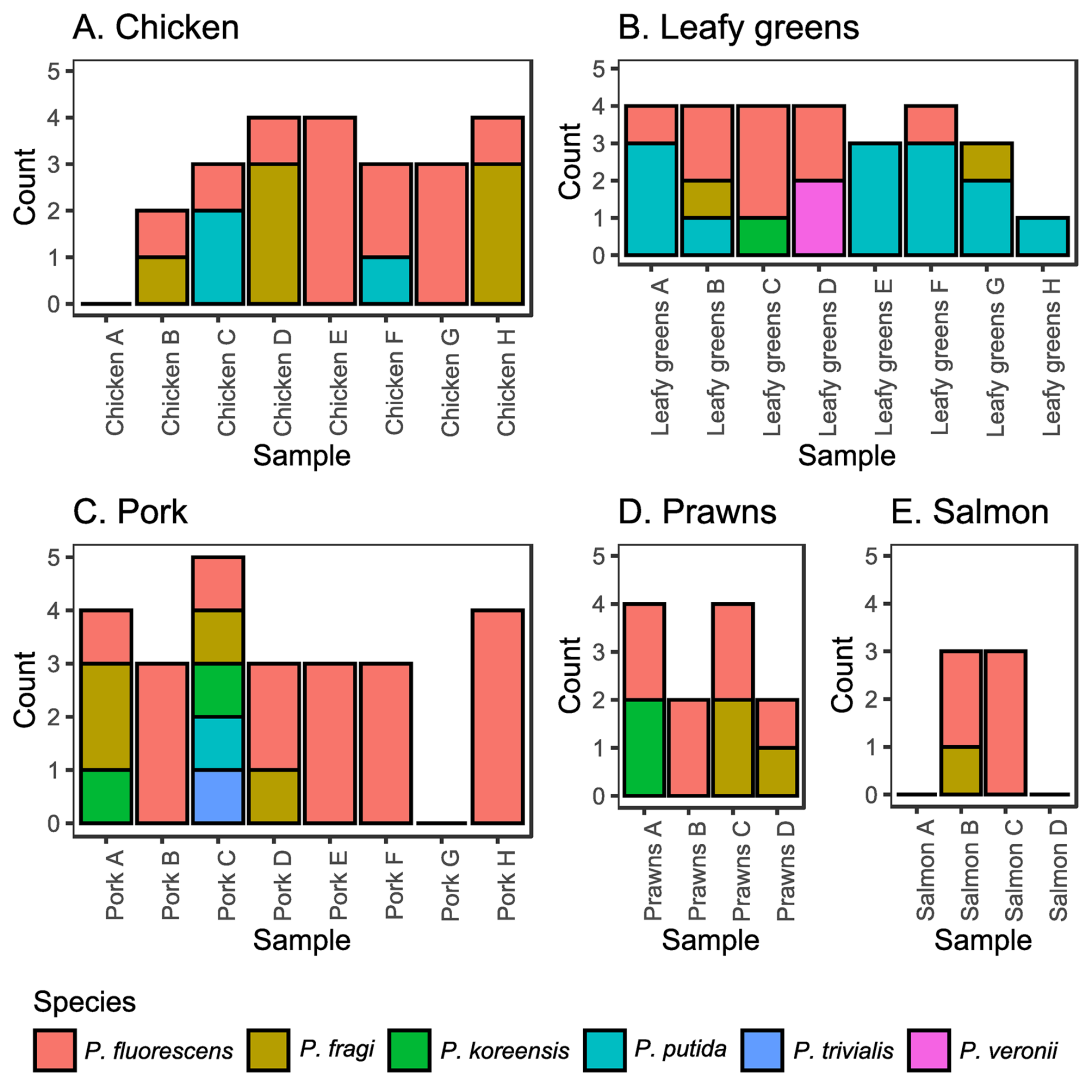
*Pseudomonas* species isolated from different food commodities. Number of *Pseudomonas* isolates analyzed from each food commodity, colored by species

The *Pseudomonas* species cultured from food belonged to the *Pseudomonas fluorescens*, *P. fragi*, *P. koreensis, P. putida, P. trivialis* and *P. veronii* species and their presence on different food commodities (Table 1) were similar to that observed in previous studies (Supplementary Table 2). However, *P. aeruginosa* was not isolated from any of these food samples using this methodology, which utilized the ISO 13720 standard. There was species diversity amongst isolates collected from the same sample, with two different *Pseudomonas* species isolated from 15 samples, three different *Pseudomonas* species isolated from two samples, and five different *Pseudomonas* species isolated from one sample (Fig. 1). There was also diversity within species, with 15 samples containing isolates that belonged to different phylogenetic clades within the same species (Supplementary Figs. 1–4).

**Table 1:** Number of food samples from each commodity that cultured for different *Pseudomonas* species

Commodity	*P. fluorescens*	*P. fragi*	*P. koreensis*	*P. putida*	*P. trivialis*	*P. veronii*	Total samples
Chicken	7	3	0	2	0	0	8
Leafy greens	5	2	1	6	0	1	8
Pork	7	3	2	1	1	0	8
Prawns	4	2	1	0	0	0	4
Salmon	2	1	0	0	0	0	4

Of the 93 *Pseudomonas* spp. genomes analyzed, none contained known ARGs or plasmid replicons. Two *P. fragi* genomes contained five virulence genes (*mrkA*, *mrkB*, *mrkC*, *mrkD* and *mrkF*); virulence genes were not identified in the other *Pseudomonas* spp. genomes. The PATRIC database yielded 33 *P. fluorescens*, 10 *P. koreensis* and 17 *P. putida* genomes; similar results were obtained with these publicly available genomes, with only a single *P. fluorescens* genome containing a plasmid replicon and four *P. putida* isolates containing ARGs.

### *P. aeruginosa* analysis

**Fig. 2:**
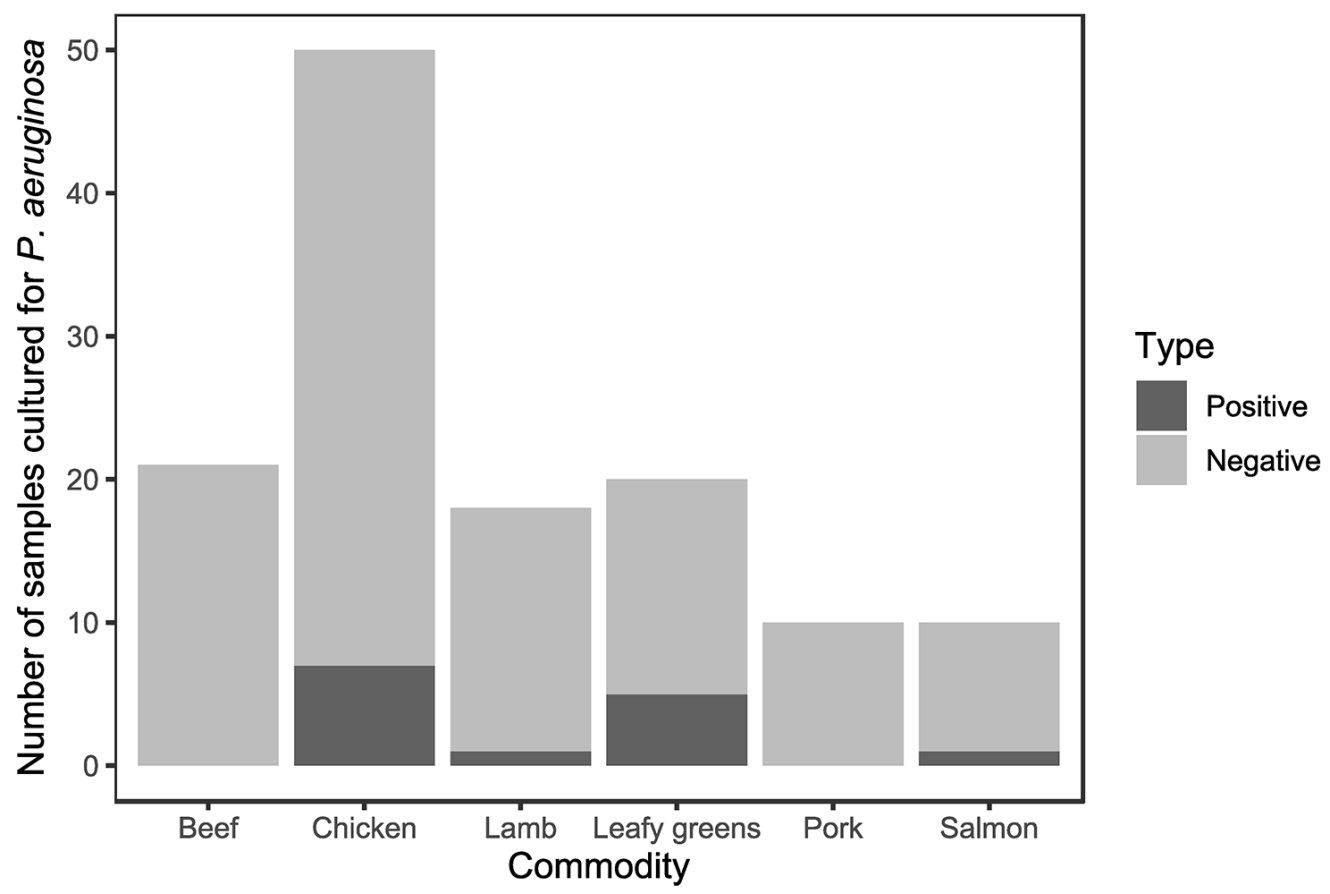
Prevalence of *Pseudomonas aeruginosa* found on different food commodities. Number of food samples belonging to each commodity that were cultured for and tested positive for *P. aeruginosa*

As we were unable to isolate *P. aeruginosa* using non-specific *Pseudomonas* spp. culturing, we specifically cultured for this bacterial species by increasing the incubation temperature and using a different medium. *P. aeruginosa* presence was evaluated in 128 food samples and was isolated from 0/21 beef, 7/50 chicken, 1/18 lamb, 5/19 leafy greens, 0/10 pork and 1/10 salmon samples (Fig. 2, Supplementary Table 3), but these proportions were not significantly different (Fisher’s exact test: *p* = 0.101). Of the 19 leafy green samples tested for *P. aeruginosa*, 13 were labelled as “washed”, three were labelled were labelled as “wash before use”, and for three it was unknown. *P. aeruginosa* was only cultured from washed leafy green samples.

**Fig. 3:**
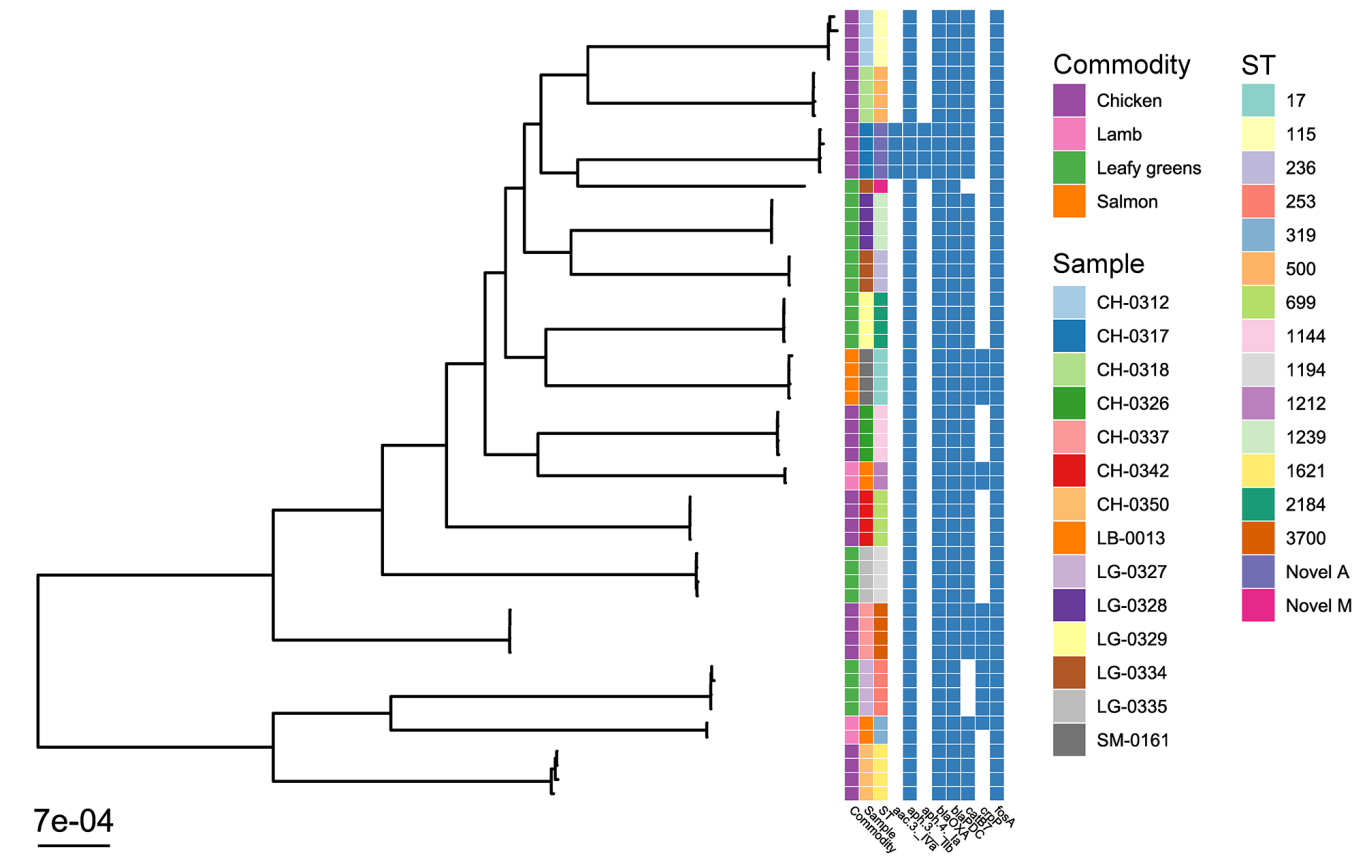
Phylogenetic relationship between *P. aeruginosa* isolates collected from retail food samples. Maximum likelihood tree of the 56 *P. aeruginosa* isolates collected from food samples, colored by sample, food type (commodity) and sequence type (ST), along with a presence-absence matrix of the antimicrobial resistance genes (ARGs) they contained. The phylogenetic branch lengths are given in nucleotide substitutions per site, therefore a branch of length 0.0007 (as represented by the scale bar) equates to 3,356 substitutions, given that the core gene alignment consisted of 4,794,505 bp

The 56 *P. aeruginosa* isolates recovered belonged to 16 unique STs, but two of the STs had not been described before so were given the temporary names Novel-A and Novel-M (Supplementary Table 4). For one lamb and one leafy green sample, two STs were identified, whilst for the remaining twelve positive samples only a single ST was identified (Fig. 3).

**Fig. 4:**
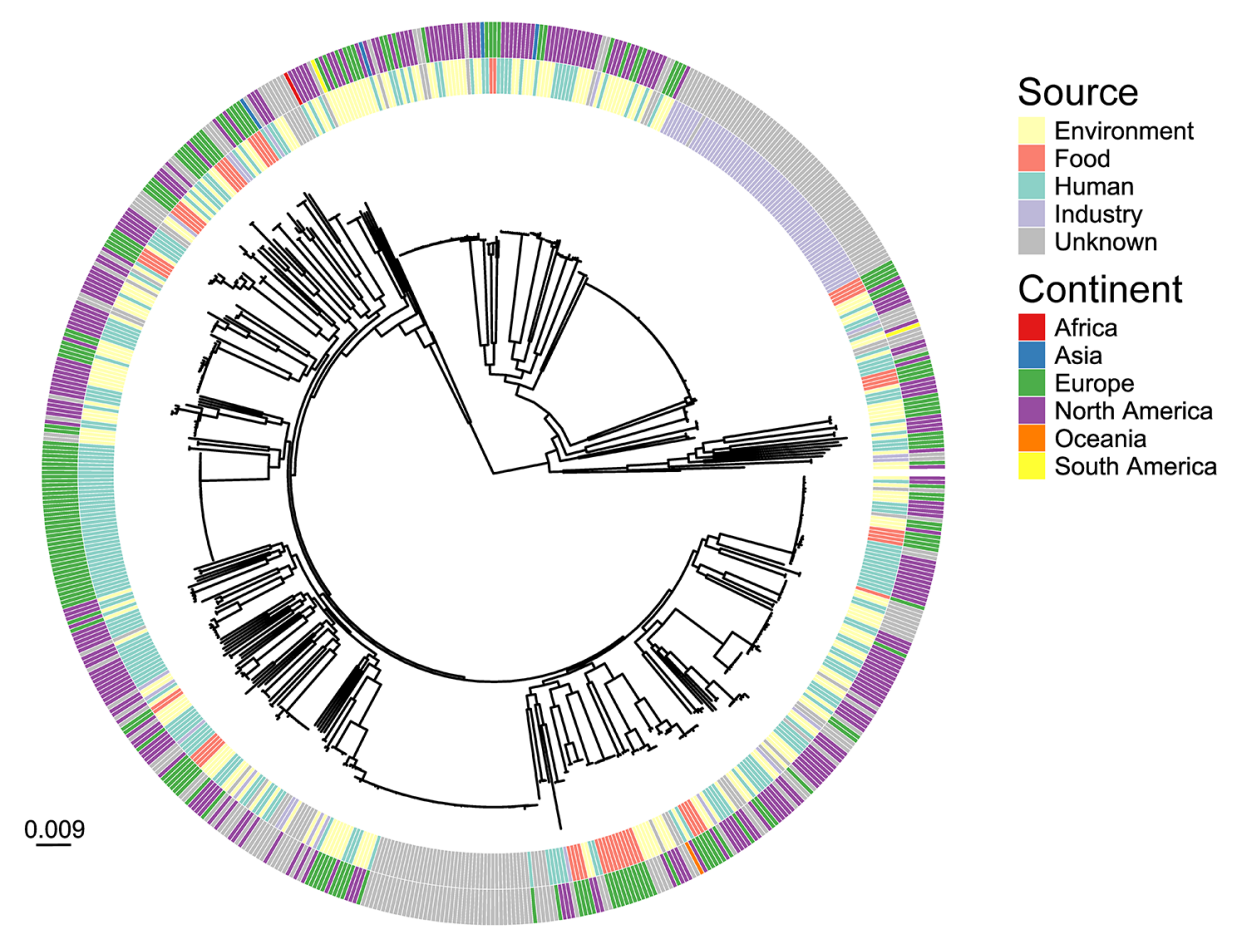
Phylogenetic relationship between *Pseudomonas aeruginosa* isolates collected from retail food samples and publicly available genomes. Maximum likelihood tree of 662 *P. aeruginosa* isolates, colored by source and continent of origin. The phylogenetic branch lengths are given in nucleotide substitutions per site, therefore a branch of length 0.009 (as represented by the scale bar) equates to 26,141 substitutions, given that the core gene alignment consisted of 2,904,504 bp

The 56 food-derived *P. aeruginosa* were compared to 606 publicly available genomes of the same species for context (Fig. 4). These isolates were mostly collected from human, industrial and environmental sources, predominantly from Europe and North America. This analysis showed food isolates were similar to isolates across the phylogeny of the context panel. All food isolates of *P. aeruginosa* in this study contained *aph(3)_IIb*, *blaOXA*, *blaPDC*, and *fosA* genes at similar frequencies to the context isolates: 99.7%, 99.5%, 99.0% and 84.2%, respectively. All food isolates contained the *catB7* gene, except for those belonging to ST-253 and novel ST-M; 84.7% of the context *P. aeruginosa* genomes investigated contained this gene. Food isolates belonging to ST-17, ST-236, ST-253, ST-3700 and one of the ST-319 isolates contained the *crpP* genes, as did 61% of the context *P. aeruginosa* investigated. *P. aeruginosa* from one food sample belonging to ST-1212 contained the *aac(3)_IVa* and *aph(4)_Ia* genes, whilst none of the context *P. aeruginosa* contained these ARGs.

None of the *P. aeruginosa* from food contained any known plasmid replicons, whilst only 2.81% of the 606 context *P. aeruginosa* genomes did so.

Amongst the 56 food and 606 context *P. aeruginosa* genomes, 239 virulence genes were identified. Individual isolates from food contained 192–231 virulence genes, similar to the context *P. aeruginosa* investigated: 152–236. There was a large amount of variation in the presence of virulence genes found amongst *P. aeruginosa* from each of the sources. The proportion of human and food isolates that contained each virulence gene were compared (Supplementary Fig. 5); the *pchE, pchG*, *pchH, ppkA, pscH* and *tagF/pppB* genes were associated with *P. aeruginosa* isolates from humans, whilst *mucA, pvdM* and *pvdN* genes were associated with *P. aeruginosa* from food (Supplementary Table 5).

### *P. aeruginosa* within-sample diversity

All *P. aeruginosa* isolates collected from the same food sample belonged to one or two STs; isolates belonging to the same ST and sample were not identical, differing by up to 19–67 core non-recombinant SNPs and 5–29 virulence genes (Table 2). 71 virulence genes varied amongst isolates belonging to the same ST and sample.

**Table 2:** Diversity of *P. aeruginosa* isolates collected from the same food sample

				SNP		Virulence	
Sample	Type	ST	Isolates	Minimum	Maximum	Minimum	Maximum
CH-0312	Chicken	115	4	9	26	9	29
CH-0317	Chicken	Novel A	4	5	25	9	26
CH-0318	Chicken	500	4	4	22	6	19
CH-0326	Chicken	1144	4	13	32	6	12
CH-0337	Chicken	3700	4	3	19	2	10
CH-0342	Chicken	699	4	0	23	4	9
CH-0350	Chicken	1621	4	33	67	6	10
LB-0013	Lamb	1212	2	19	19	5	5
LB-0013	Lamb	319	2	54	54	6	6
LG-0327	Leafy greens	253	4	7	14	4	7
LG-0328	Leafy greens	1239	4	8	25	2	6
LG-0329	Leafy greens	2184	4	11	28	2	6
LG-0334	Leafy greens	Novel M	1	-	-	-	-
LG-0334	Leafy greens	236	3	5	7	3	6
LG-0335	Leafy greens	1194	4	16	28	3	6
SM-0161	Salmon	17	4	1	34	3	19

### *P. aeruginosa* clinical isolates

Of the 16 STs within the 56 food *P. aeruginosa* genomes, publicly available genomes from human samples were available for four: ST-17, ST-253, ST-319 and ST-699. The minimum number of core non-recombinant SNPs between the isolates from human and food samples for these STs was 41–312 (Supplementary Table 6). The most closely related human isolates differed by 0–1 ARGs, no plasmid replicons and 3–6 virulence genes, and were collected between 1991 and 1997 from France and Turkey, or this information was not available.

### Virulence of *P. aeruginosa* using the *Galleria mellonella* larvae infection model

Results from the *Galleria mellonella* larvae infection model varied between replicates (Supplementary Fig. 6; Supplementary Table 7). Of the 16 *P. aeruginosa* STs collected from food 15, were more virulent than the low virulence control (PAO1) (Supplementary Fig. 6); these differences were not statistically significant (Supplementary Table 8). None of the strains isolated were as virulent as the high virulence control (PA14).

## Discussion

The *Pseudomonas* genus has been described as one of the most ubiquitous bacterial genera found in environmental, human and animal sources globally [19]. This was evident when we cultured 88% of retail food samples for this bacterial genus, but did not identify any isolates belonging to the *P. aeruginosa* species, suggesting that although *Pseudomonas* make up a large portion of the food metagenome [4], *P. aeruginosa* is not a large contributor. The six other *Pseudomonas* species isolated from food varied in the types of food from which they were isolated (Supplementary Table 2). There were some differences to previous studies; for examples, we isolated *P. koreensis* from prawns, and, to the best of our knowledge, this is the first time this species has been isolated from seafood; we also failed to isolate *P. putida* from any seafood samples or *P. trivialis* from any leafy greens, even though they have previously been reported from similar sources [20, 21]. However, we only examined 32 food samples with the ISO 13720 standard method, and may find particular *Pseudomonas* species on other food types with further sampling.

The risk of human infection varies by *Pseudomonas* species. *P. fluorescens* has been associated with infections, but usually these are in immunocompromised patients associated with contaminated pharmaceuticals [22]. *P. koreensis* was previously isolated from a keratitis case but was part of a mixed infection with *Aspergillus fumigatus*, likely the result of a contaminated contact lens [23]. *P. putida* has been isolated from bloodstream infections, but these were assumed to be nosocomial infections [24]. To the best of our knowledge, *P. fragi*, *P. trivalis* and *P. veronii* have not been isolated from any clinical samples. Therefore, the presence of these non-*aeruginosa Pseudomonas* species on food is unlikely to be a direct health concern. Furthermore, they did not contain any known ARGs. This suggests that although the non-*aeruginosa Pseudomonas* comprise a large proportion of the bacteria found on food, they are unlikely to act as a major reservoir of ARGs for pathogens.

The potential impacts of non-*aeruginosa Pseudomonas* spp. on food goes beyond clinical concerns; the genus is recognized as a major cause of food spoilage [25]. Of the *Pseudomonas* species identified in this study, *P. fluorescens* is the best described cause of food spoilage, having been associated with spoilage in a wide range of food types from fruits and vegetables, dairy products, meat and seafood [26]. In addition, *P. fragi, P. koreensis* and *P. putida* have also been found to cause the spoilage of specific meats and seafoods [27, 28]. A *mrk* gene cluster was identified in two *P. fragi* genomes cultured from food; this gene cluster originates from *Klebsiella* and encodes type III fimbriae that are used for cell adhesion and biofilm formation [29]. In Enterobacterales, the *mrk* gene cluster is associated with increased biofilm formation and isolates from catheter-associated urinary tract infections [30]. *P. fragi* has not been isolated from clinical samples, but improved biofilm formation could facilitate persistence during food processing. The high prevalence and diverse populations of these bacteria on the food types described in this study highlights the need to identify how food becomes contaminated with *Pseudomonas*, what food processing techniques facilitate or prevent their contamination, and how the relative proportions of the *Pseudomonas* species change over the shelf-life of food.

*Pseudomonas* spp. was isolated from 88% of food samples using the ISO 13720 standard method, but *P. aeruginosa* isolation required different culture conditions, including raising the incubation temperature from 25 °C to 37 °C, a 24-hour enrichment step and a more selective medium (*Pseudomonas* centrimide (CN) instead of *Pseudomonas* cephalothin-sodium fusidate-cetrimide (CFC) agar). Despite these adjustments, *P. aeruginosa* was still only isolated from 11% of food samples. *P. aeruginosa* seems to make up a small proportion of the *Pseudomonas* spp. found on food, if it is present at all. It is possible that *P. aeruginosa* could have been recovered using the ISO 13720 standard method if we had analyzed more isolates from each sample. However, the number required to reach the sensitivity of the CN agar-37 °C method remains to be determined.

*P. aeruginosa* has been isolated from a wide range of human clinical samples [8–10], but source of infections are hard to identify due to the ubiquitous nature of the bacterium [12]. Wheatley et al. [17] demonstrated that *P. aeruginosa* from the gastrointestinal tract could travel to colonize the lungs of a patient, showing that carrying a reservoir of *P. aeruginosa* in the gut can be a risk factor for serious disease. Although *P. aeruginosa* has been found in public water supplies, representing one route of transmission [31], our data suggest gastrointestinal colonization could also be due to contaminated food as we isolated *P. aeruginosa* from 11% of food samples of different types. Four out of 16 *P. aeruginosa* STs isolated were within 41–312 SNPs to an isolate from a human clinical sample [32–35]. This indicates that humans are regularly exposed to *P. aeruginosa* isolated from food and this exposure has the potential to lead to opportunistic infections in vulnerable people.

The *G. mellonella* larvae model is a simple model for investigating *P. aeruginosa* virulence [36]. *P. aeruginosa* kills *G. mellonella* larvae very effectively, particularly in comparison to other pathogens [37]. For this reason, approximately three *P. aeruginosa* cells were inoculated into each larva. There were no significant differences in larvae survivability between the *P. aeruginosa* strains isolated from food and the low virulence control strain, but there was variation in survivability between replicates. More consistent methods are required to compare the virulence of these *P. aeruginosa* strains.

*P. aeruginosa* was isolated from chicken, lamb, leafy greens and salmon samples. No beef or pork samples were positive, but further sampling may identify *P. aeruginosa* from these commodities. Wong et al. [38] cultured eight chicken and eight pork samples for meropenem-resistant *Pseudomonas*, but only isolated *P. aeruginosa* from one of the pork samples. Most of the food samples investigated in this study consisted of meat and seafood samples that are likely to be cooked prior to consumption. However, leafy greens were the food commodity with the highest proportion of *P. aeruginosa* isolates, and they are unlikely to be further processed prior to consumption, especially as the five leafy green samples from which *P. aeruginosa* was cultured were already washed and one of these samples contained an ST previously identified in human clinical samples. Further studies are required to investigate *P. aeruginosa* from a wider range of food commodities to obtain better insight into which food types are most frequently contaminated with the bacterium, if the *P. aeruginosa* isolated are associated with clinical infections and what factors influence its prevalence on food, such as geographical origin, cut of meat and storage temperature.

The collection of multiple *P. aeruginosa* isolates from each food samples helped determine within-sample bacterial diversity. Multiple STs were collected from two samples, and isolates belonging to the same ST and sample differed by up to 19–67 core non-recombinant SNPs, along with variable presence of ARGs and virulence genes. Most *P. aeruginosa* strains have a substitution rate of 4.3 × 10^− 6^ – 1.0 × 10^− 5^ substitutions site^− 1^ year^− 1^, equating to 27–63 SNPs per year, although strains with faster substitution rates have been described [39]. Therefore, it would take 3 to 30 months for isolates collected from the same food sample to accumulate the number of SNPs identified. This is possible with salmon that are usually harvested after 24 months [40] and lamb that are usually slaughtered at 2–6 months [41], but not leafy greens such as lettuce that are usually harvested after 2–3 months [42] or chicken that is usually slaughtered at 1–2 months [43]. Therefore, for leafy greens and chicken samples, and samples with multiple STs identified, they were likely contaminated with a heterogenous population of *P. aeruginosa* or were contaminated with *P. aeruginosa* at multiple time points. This complicates *P. aeruginosa* source attribution studies as multiple isolates will need to be collected from food to capture ST and SNP diversity.

*P. aeruginosa* contain multiple virulence genes that allow them to cause opportunistic infections. These include genes involved in biofilm formation, quorum sensing, intracellular survival, and acquiring nutrients [44]. We identified three virulence genes associated with a larger proportion of food isolates compared to human isolates: *mucA, pvdM* and *pvdN*. *mucA* encodes a negative regulator of alginate production, and *mucA* mutations are associated with a mucoid *P. aeruginosa* phenotype [45]. In cystic fibrosis patients, mucoidal *P. aeruginosa* are often selected for via inactivation of the *mucA* gene [46], which could explain why a smaller proportion of human isolates contained an intact version of this gene compared to food isolates, as it is likely that many human clinical isolates were derived from cystic fibrosis patients. *pvdM* encodes an enzyme essential for the production of the siderophore pyoverdine [47], whilst *pvdN* encodes an enzyme that modifies pyoverdine, but the effect of this modification is not clear [48]. O’Brien et al. [49] investigated *P. aeruginosa* from long-term cystic fibrosis patients and found many have subpopulations of this bacterium with reduced pyoverdine. Therefore, the lack of the *pvdM* and *pvdN* genes in many human isolates may have been selected for in chronic infections. In addition, six virulence genes were identified that were found in a larger proportion of human isolates than food: *pchE, pchG, pchH*, *ppkA*, *pscH* and *tagF/pppB*. *pchE* [50] and *pchG* [51] encode enzymes essential for the production of pyochelin, a siderophore that contributes to pathogenicity, whilst *pchH* is found in the same operon as these genes but its exact function is unknown. *ppkA* encodes a serine/threonine protein kinase and knockout studies found it is associated with biofilm formation, pyocyanin production, tolerance to oxidative and osmotic stresses, and host cell invasion [52]. *pscH* encodes a protein involved in the type III secretory system of *P. aeruginosa* involved in virulence by injecting effector proteins into host cells [53]. *tagF*/*pppB* regulates the type VI secretion system that is used to deliver effector proteins into eukaryotic host cells or bacterial competitors [54]. *P. aeruginosa* are involved in a wide range of opportunistic infections, and the lack of these virulence genes in many food isolates may prevent them forming certain opportunistic infections if given the chance.

*P. aeruginosa* infections are difficult to treat due to their large number of inherent and acquired AMR mechanisms [11]. These mechanisms were evident with those collected from food, where all isolates contained *aph(3)_IIb*, *blaOXA_50*, *blaPDC*, and *fosA* genes, whilst they varied in the presence of the *aac(3)_IVa*, *aph(4)_Ia, catB7* and *crpP* genes, similarly to the other *P. aeruginosa* genomes investigated. However, as no *P. aeruginosa* were isolated when non-specifically culturing for *Pseudomonas* and the *Pseudomonas* species that were contained no ARGs, the *Pseudomonas* genus does not likely contribute much to the ARG reservoir of food.

## Conclusions

*P. aeruginosa* isolates likely to be able to cause human infections are present on a relatively small but still substantial proportions of retail foods examined. Whilst the *P. aeruginosa* isolates contained a multidrug resistant genotype, the most frequent non-*P. aeruginosa* recovered carried no ARGs and are more likely to play a role in food spoilage rather than infection. Given the frequency of exposure, the potential contribution of food to the burden of *P. aeruginosa* infections in humans should be evaluated more closely.

## Materials and methods

### *Pseudomonas* spp. culturing

Retail food samples used for *Pseudomonas* spp. culturing were collected as part of a previously reported repeated cross-sectional study of retail food in Norfolk, UK [55]. Chicken (*n* = 8), leafy greens (*n* = 8), pork (*n* = 8), prawn (*n* = 4) and salmon (*n* = 4) products were collected on 25/11/2019. *Pseudomonas* spp. were cultured from these samples using a method adapted from ISO 13720 [56]. For each food sample, 100 g was aseptically transferred into a sterile filtered stomacher bag (Corning, New York, USA). All sample types were homogenized in 225 ml of buffered peptone water (BPW) at 100 rpm for 30 s (Seward stomacher 400 C laboratory blender, Worthing, UK). For samples containing bones or shells, homogenization was performed manually for two minutes. A 10 µl loopful of stomached food was inoculated onto CFC agar (Oxoid, Basingstoke, UK) and streaked for single colonies. Inoculated CFC agar plates were incubated at 25 °C for 48 h. Eight colonies were subcultured onto separate tryptic soy agar (TSA) plates (Trafalgar Scientific Ltd., Leicester, UK), streaked for single colonies and incubated at 25 °C for 48 h. An oxidase test (Oxoid) was performed on all colonies on TSA and up to five of those that tested positive underwent WGS to confirm they were *Pseudomonas* spp.

### *P. aeruginosa* culturing

Retail food samples used for specific *P. aeruginosa* culturing were collected in Norwich, Norfolk, UK. Beef (*n* = 21), chicken (*n* = 50), lamb (*n* = 18), leafy greens (*n* = 19), pork (*n* = 10) and salmon (*n* = 10) were collected between 14/03/2021-30/09/2022. 100 g of each food sample was aseptically transferred into a sterile filtered stomacher bag. All sample types were homogenized in 225 mL of BPW at 100 rpm for 30 s. Stomached food samples were pre-enriched by incubating them at 37 °C for 24 h in BPW. A 10 µL loopful of pre-enriched food was inoculated onto *Pseudomonas* CN agar (Oxoid) and streaked for single colonies. Inoculated *Pseudomonas* CN agar plates were incubated at 37 °C for 48 h. Four colonies morphologically consistent with being *P. aeruginosa* (producing green/blue pigment) were subcultured onto TSA plates (Trafalgar Scientific Ltd.) and streaked for single colonies, before being incubated at 37 °C for 24 h. An oxidase test (Oxoid) was performed on all colonies cultured on TSA and isolates that tested positive were assumed to be *P. aeruginosa*. WGS was used to confirm *P. aeruginosa* identity.

### Whole genome sequencing

DNA was extracted using the Maxwell® RSC Cultured Cells DNA Kit (Promega, Madison, Wisconsin, USA) using the manufacturer’s instructions. Libraries were created using the Nextera XT DNA Library Preparation Kit (Illumina, San Diego, California, USA) and sequenced on a NextSeq 550 System (Illumina) as 2 × 150 bp paired-end reads.

### Genomic analysis

Genomic analyses were performed using the Cloud Infrastructure for Microbial Bioinformatics (CLIMB) [57]. Raw paired-end reads were trimmed using Trimmomatic v0.36 [58] (Supplementary material) and assembled using Spades v3.11.1 [59] in “careful” mode. Centrifuge v1.0.3 [60] was used to predict *Pseudomonas* species. The quality of genome assemblies was assessed using QUAST v4.6.3 [61], CheckM v1.1.2 [62] and by aligning reads to the assemblies using the Burrows-Wheeler aligner (BWA) v0.7.17 [63]. Assemblies were accepted if they consisted of less than 500 contigs that were over 500 bp, less than 50 duplicate genes and had a mean read depth of the four largest contigs above 30. ARGs, virulence genes and plasmid replicons were identified using ARIBA v2.14.4 [64] and the NCBI AMR [65], virulence finder database (VFDB) [66] and PlasmidFinder [67] databases, respectively.

### Context collection of *Pseudomonas* spp.

The PATRIC database [68] was searched for genomes belonging to the *Pseudomonas* species identified in this study. These genomes were downloaded and the quality checked. Those that passed QC had ARGs, plasmid replicons and virulence genes identified. To compare *Pseudomonas* spp. genomes belonging to the same species, the genomes were annotated using Prokka v1.13 [69], Roary v3.11.2 [70] was used to cluster the genes of each genome using a 95% identity cut-off and classifying genes that were found in 95% of isolates as core. RAxML v8.2.4 [71] was used to form a maximum likelihood tree from the core gene alignments of each species using a generalized-time reversible (GTR) substitution model [72]. TreeCluster v1.0.3 [73] was used to predict clades from the maximum likelihood trees for these species using a 0.02 length threshold.

### *P. aeruginosa* sequence type analysis

The sequence types (STs) of *P. aeruginosa* genomes were predicted in silico using MLST v2.16.1 (https://github.com/tseemann/mlst). Single nucleotide polymorphisms (SNPs) were identified using an alignment-based approach using NC_002516 as the reference genome. Phaster [74] was used to identify prophage regions in the reference genome and phage regions were blocked out. Snippy v3.1 (https://github.com/tseemann/snippy) was used to align *P. aeruginosa* trimmed reads from each ST to the prophage-free reference. Gubbins v2.3.1 [75] was used to remove SNPs putatively associated with recombination and RAxML was used to generate a maximum likelihood tree based on non-recombinant SNPs using a GTR substitution model.

### Virulence of *P. aeruginosa* using the *Galleria mellonella* larvae infection model

The *Galleria mellonella* larvae infection model has been used previously to investigate the virulence of *P. aeruginosa* [76]. Here, it was used to compare 16 selected food isolates to known virulent control strains PA14 and PAO1 [77]. Preliminary investigations determined the LD50 (lethal dose required to kill 50% of larvae per replicate after 48 h) of the less virulent control strain PAO1 was approximately three colony-forming units (CFU) per inoculum. Fresh bacterial cultures were normalized to an optical density of 0.1 at 600 nm and diluted in sterile phosphate-buffered saline (PBS) (Sigma-Aldrich, St Louis, MO, USA) to achieve the LD50 inoculum concentration. The inoculum concentrations were confirmed by plating 50 µL of inoculum on Luria-Bertani (LB) agar (ThermoFisher Scientific, Waltham, MA, USA), incubating the plates at 37 °C and counting the colonies the following day. The wax moth larvae (Livefood UK, Rooks Bridge, UK) chosen for infection were similarly sized with no signs of pupation or melanization. Each larva was injected with 10 µL of inoculum using a 10 µL syringe (Hamilton, Reno, NV, USA) into the left penultimate proleg. Syringes were sterilized with 70% ethanol (VWR International, Radnor, PA, USA) and washed with sterile PBS between each infection. Each strain was injected into 10 larvae and the percentage of surviving larvae was determined every 3–17 h over 48 h. Controls included ten larvae injected with sterile PBS only and ten uninjected larvae. The infection model was repeated on three independent occasions.

### Statistics and reproducibility

Fisher’s exact test was used to determine if there were any associations between the proportion of samples that cultured positive for *P. aeruginosa* and food commodity.

For each virulence gene, the proportion of *P. aeruginosa* isolates from human and food sources that contained them were calculated. Two-population t-tests were used to determine if the proportions were significantly different. Bonferroni’s correction for multiple hypothesis testing was used to take into consideration the total number of virulence genes investigated (*p* = 0.0002).

The survivorship of *G. mellonella* larvae was modelled using a mixed-model logistic regression model using the package glmmTMB v1.1.8 [78] in R v4.1.2 [79], with strain and replicate as fixed effects and the interaction between run and strain as a random effect.

### Electronic supplementary material

Below is the link to the electronic supplementary material.

Supplementary Material 1

## Data Availability

The sequence data generated during the current study are available in the Sequence Read Archive under Bioproject: PRJNA973713.

## List of Abbreviations

AMR: Antimicrobial resistance
ARG: Antimicrobial resistance gene
BPW: Buffered peptone water
BWA: Burrows–Wheeler aligner
CFC: Cephalothin–sodium fusidate–cetrimide
CLIMB: Cloud Infrastructure for Microbial Bioinformatics
CN: Centrimide
*G. mellonella*: *Galleria mellonella*
GTR: Generalized time reversible
MLST: Multilocus sequence typing
*P. aeruginosa*: *Pseudomonas aeruginosa*
*P. fluorescens*: *Pseudomonas fluorescens*
*P. fragi*: *Pseudomonas fragi*
*P. koreensis*: *Pseudomonas koreensis*
*P. putida*: *Pseudomonas putida*
*P. trivialis*: *Pseudomonas trivialis*
*P. veronii*: *Pseudomonas veronii*
PBS: Phosphate buffered saline
QC: Quality control
SNP: Single nucleotide polymorphism
SRA: Sequence read archive
ST: Sequence type
