# Supplementary material for: Genomic characterization of Pseudomonas spp. on food: implications for spoilage, antimicrobial resistance and human infection
Source: BMC Microbiol. 2024 Jan 11;24:20. doi: 10.1186/s12866-023-03153-9 (PMC10782663; doi:10.1186/s12866-023-03153-9)
Supplement: Supplementary file 1 — Supplementary Material 1 [file 12866_2023_3153_MOESM1_ESM.docx]

**Read trimming command**

Raw paired reads were trimmed using the following Trimmomatic command:

trimmomatic PE -threads <number of threads> -phred33 <forward read file> < reverse read file> <forward paired read outfile> <forward unpaired read outfile> <reverse paired read outfile> <reverse unpaired read outfile> ILLUMINACLIP:<library adapter file>:2:30:10 LEADING:3 TRAILING:3 SLIDINGWINDOW:4:25 MINLEN:50.

| **Table S1**. Metadata of *Pseudomonas* spp. isolates cultured from food. | | | | |  |
| --- | --- | --- | --- | --- | --- |
| Isolate | Sample | Species | Commodity | Renamed sample | Accession |
| PS18CH-0305-5 | CH-0305 | *P. fluorescens* | Chicken | Chicken B | SRR24626084 |
| PS18CH-0305-7 | CH-0305 | *P. fragi* | Chicken | Chicken B | SRR24625987 |
| PS18CH-0306-1 | CH-0306 | *P. putida* | Chicken | Chicken C | SRR24626083 |
| PS18CH-0306-2 | CH-0306 | *P. fluorescens* | Chicken | Chicken C | SRR24625986 |
| PS18CH-0306-5 | CH-0306 | *P. putida* | Chicken | Chicken C | SRR24626023 |
| PS18CH-0307-1 | CH-0307 | *P. fragi* | Chicken | Chicken D | SRR24626012 |
| PS18CH-0307-3 | CH-0307 | *P. fluorescens* | Chicken | Chicken D | SRR24626001 |
| PS18CH-0307-5 | CH-0307 | *P. fragi* | Chicken | Chicken D | SRR24625990 |
| PS18CH-0307-6 | CH-0307 | *P. fragi* | Chicken | Chicken D | SRR24625985 |
| PS18CH-0308-1 | CH-0308 | *P. fluorescens* | Chicken | Chicken E | SRR24625958 |
| PS18CH-0308-3 | CH-0308 | *P. fluorescens* | Chicken | Chicken E | SRR24625947 |
| PS18CH-0308-5 | CH-0308 | *P. fluorescens* | Chicken | Chicken E | SRR24625936 |
| PS18CH-0308-7 | CH-0308 | *P. fluorescens* | Chicken | Chicken E | SRR24625984 |
| PS18CH-0309-1 | CH-0309 | *P. fluorescens* | Chicken | Chicken F | SRR24625978 |
| PS18CH-0309-2 | CH-0309 | *P. fluorescens* | Chicken | Chicken F | SRR24626082 |
| PS18CH-0309-3 | CH-0309 | *P. putida* | Chicken | Chicken F | SRR24626071 |
| PS18CH-0310-1 | CH-0310 | *P. fluorescens* | Chicken | Chicken G | SRR24626060 |
| PS18CH-0310-3 | CH-0310 | *P. fluorescens* | Chicken | Chicken G | SRR24626049 |
| PS18CH-0310-5 | CH-0310 | *P. fluorescens* | Chicken | Chicken G | SRR24626038 |
| PS18CH-0311-1 | CH-0311 | *P. fragi* | Chicken | Chicken H | SRR24626028 |
| PS18CH-0311-3 | CH-0311 | *P. fragi* | Chicken | Chicken H | SRR24626027 |
| PS18CH-0311-4 | CH-0311 | *P. fluorescens* | Chicken | Chicken H | SRR24625983 |
| PS18CH-0311-5 | CH-0311 | *P. fragi* | Chicken | Chicken H | SRR24626026 |
| PS18LG-0304-1 | LG-0304 | *P. putida* | Leafy greens | Leafy greens A | SRR24626025 |
| PS18LG-0304-3 | LG-0304 | *P. fluorescens* | Leafy greens | Leafy greens A | SRR24626024 |
| PS18LG-0304-5 | LG-0304 | *P. putida* | Leafy greens | Leafy greens A | SRR24626022 |
| PS18LG-0304-7 | LG-0304 | *P. putida* | Leafy greens | Leafy greens A | SRR24625982 |
| PS18LG-0305-1 | LG-0305 | *P. fluorescens* | Leafy greens | Leafy greens B | SRR24626021 |
| PS18LG-0305-3 | LG-0305 | *P. fluorescens* | Leafy greens | Leafy greens B | SRR24626020 |
| PS18LG-0305-5 | LG-0305 | *P. putida* | Leafy greens | Leafy greens B | SRR24626019 |
| PS18LG-0305-7 | LG-0305 | *P. fragi* | Leafy greens | Leafy greens B | SRR24625981 |
| PS18LG-0306-1 | LG-0306 | *P. fluorescens* | Leafy greens | Leafy greens C | SRR24626018 |
| PS18LG-0306-3 | LG-0306 | *P. fluorescens* | Leafy greens | Leafy greens C | SRR24626017 |
| PS18LG-0306-5 | LG-0306 | *P. fluorescens* | Leafy greens | Leafy greens C | SRR24626016 |
| PS18LG-0306-8 | LG-0306 | *P. koreensis* | Leafy greens | Leafy greens C | SRR24625980 |
| PS18LG-0307-1 | LG-0307 | *P. fluorescens* | Leafy greens | Leafy greens D | SRR24626015 |
| PS18LG-0307-2 | LG-0307 | *P. fluorescens* | Leafy greens | Leafy greens D | SRR24626014 |
| PS18LG-0307-3 | LG-0307 | *P. veronii* | Leafy greens | Leafy greens D | SRR24626013 |
| PS18LG-0307-4 | LG-0307 | *P. veronii* | Leafy greens | Leafy greens D | SRR24625979 |
| PS18LG-0308-1 | LG-0308 | *P. putida* | Leafy greens | Leafy greens E | SRR24626011 |
| PS18LG-0308-3 | LG-0308 | *P. putida* | Leafy greens | Leafy greens E | SRR24626010 |
| PS18LG-0308-5 | LG-0308 | *P. putida* | Leafy greens | Leafy greens E | SRR24626009 |
| PS18LG-0309-1 | LG-0309 | *P. putida* | Leafy greens | Leafy greens F | SRR24626008 |
| PS18LG-0309-3 | LG-0309 | *P. putida* | Leafy greens | Leafy greens F | SRR24626007 |
| PS18LG-0309-5 | LG-0309 | *P. putida* | Leafy greens | Leafy greens F | SRR24626006 |
| PS18LG-0309-6 | LG-0309 | *P. fluorescens* | Leafy greens | Leafy greens F | SRR24625977 |
| PS18LG-0310-3 | LG-0310 | *P. putida* | Leafy greens | Leafy greens G | SRR24626005 |
| PS18LG-0310-4 | LG-0310 | *P. fragi* | Leafy greens | Leafy greens G | SRR24625976 |
| PS18LG-0310-5 | LG-0310 | *P. putida* | Leafy greens | Leafy greens G | SRR24626004 |
| PS18LG-0311-4 | LG-0311 | *P. putida* | Leafy greens | Leafy greens H | SRR24625975 |
| PS18PK-0304-1 | PK-0304 | *P. koreensis* | Pork | Pork A | SRR24626003 |
| PS18PK-0304-3 | PK-0304 | *P. fragi* | Pork | Pork A | SRR24626002 |
| PS18PK-0304-5 | PK-0304 | *P. fragi* | Pork | Pork A | SRR24626000 |
| PS18PK-0304-7 | PK-0304 | *P. fluorescens* | Pork | Pork A | SRR24625999 |
| PS18PK-0305-1 | PK-0305 | *P. fluorescens* | Pork | Pork B | SRR24625998 |
| PS18PK-0305-3 | PK-0305 | *P. fluorescens* | Pork | Pork B | SRR24625997 |
| PS18PK-0305-5 | PK-0305 | *P. fluorescens* | Pork | Pork B | SRR24625996 |
| PS18PK-0306-1 | PK-0306 | *P. putida* | Pork | Pork C | SRR24625995 |
| PS18PK-0306-2 | PK-0306 | *P. koreensis* | Pork | Pork C | SRR24625994 |
| PS18PK-0306-3 | PK-0306 | *P. fragi* | Pork | Pork C | SRR24625993 |
| PS18PK-0306-5 | PK-0306 | *P. trivialis* | Pork | Pork C | SRR24625992 |
| PS18PK-0306-7 | PK-0306 | *P. fluorescens* | Pork | Pork C | SRR24625991 |
| PS18PK-0307-1 | PK-0307 | *P. fluorescens* | Pork | Pork D | SRR24625989 |
| PS18PK-0307-4 | PK-0307 | *P. fluorescens* | Pork | Pork D | SRR24625967 |
| PS18PK-0307-5 | PK-0307 | *P. fragi* | Pork | Pork D | SRR24625966 |
| PS18PK-0308-1 | PK-0308 | *P. fluorescens* | Pork | Pork E | SRR24625965 |
| PS18PK-0308-2 | PK-0308 | *P. fluorescens* | Pork | Pork E | SRR24625964 |
| PS18PK-0308-3 | PK-0308 | *P. fluorescens* | Pork | Pork E | SRR24625963 |
| PS18PK-0309-1 | PK-0309 | *P. fluorescens* | Pork | Pork F | SRR24625962 |
| PS18PK-0309-3 | PK-0309 | *P. fluorescens* | Pork | Pork F | SRR24625961 |
| PS18PK-0309-6 | PK-0309 | *P. fluorescens* | Pork | Pork F | SRR24625960 |
| PS18PK-0311-1 | PK-0311 | *P. fluorescens* | Pork | Pork H | SRR24625959 |
| PS18PK-0311-2 | PK-0311 | *P. fluorescens* | Pork | Pork H | SRR24625957 |
| PS18PK-0311-3 | PK-0311 | *P. fluorescens* | Pork | Pork H | SRR24625956 |
| PS18PK-0311-4 | PK-0311 | *P. fluorescens* | Pork | Pork H | SRR24625955 |
| PS18PR-0276-1 | PR-0276 | *P. koreensis* | Prawns | Prawns A | SRR24625954 |
| PS18PR-0276-3 | PR-0276 | *P. fluorescens* | Prawns | Prawns A | SRR24625953 |
| PS18PR-0276-4 | PR-0276 | *P. koreensis* | Prawns | Prawns A | SRR24625952 |
| PS18PR-0276-5 | PR-0276 | *P. fluorescens* | Prawns | Prawns A | SRR24625951 |
| PS18PR-0277-2 | PR-0277 | *P. fluorescens* | Prawns | Prawns B | SRR24625950 |
| PS18PR-0277-8 | PR-0277 | *P. fluorescens* | Prawns | Prawns B | SRR24625949 |
| PS18PR-0278-1 | PR-0278 | *P. fragi* | Prawns | Prawns C | SRR24625948 |
| PS18PR-0278-2 | PR-0278 | *P. fluorescens* | Prawns | Prawns C | SRR24625946 |
| PS18PR-0278-3 | PR-0278 | *P. fragi* | Prawns | Prawns C | SRR24625945 |
| PS18PR-0278-4 | PR-0278 | *P. fluorescens* | Prawns | Prawns C | SRR24625944 |
| PS18PR-0279-1 | PR-0279 | *P. fluorescens* | Prawns | Prawns D | SRR24625943 |
| PS18PR-0279-2 | PR-0279 | *P. fragi* | Prawns | Prawns D | SRR24625942 |
| PS18SM-0155-1 | SM-0155 | *P. fragi* | Salmon | Salmon B | SRR24625941 |
| PS18SM-0155-2 | SM-0155 | *P. fluorescens* | Salmon | Salmon B | SRR24625940 |
| PS18SM-0155-3 | SM-0155 | *P. fluorescens* | Salmon | Salmon B | SRR24625939 |
| PS18SM-0156-1 | SM-0156 | *P. fluorescens* | Salmon | Salmon C | SRR24625938 |
| PS18SM-0156-2 | SM-0156 | *P. fluorescens* | Salmon | Salmon C | SRR24625937 |
| PS18SM-0156-3 | SM-0156 | *P. fluorescens* | Salmon | Salmon C | SRR24625988 |

**Table S2**. *Pseudomonas* species associated with different food types in this study and in previous studies (citations in parentheses).

|  | Cultured from food in this study | | | Identified in previous studies | | |
| --- | --- | --- | --- | --- | --- | --- |
| Species | Meat | Plants | Seafood | Meat | Plants | Seafood |
| *P. fluorescens* | Chicken, Pork | Leafy greens | Prawns, Salmon | Chicken, Pork [1] | Cottonwood [2] | Gilt-head bream [3] |
| *P.*  *fragi* | Chicken, Pork | Leafy greens | Prawns, Salmon | Chicken, Pork [4] | Garlic [5] | Gilt-head bream [3] |
| *P. koreensis* | Pork | Leafy greens | Prawns, Salmon | Chicken [6] | Rice [7] |  |
| *P.*  *putida* | Chicken, Pork | Leafy  greens | | Chicken, Pork [4] | Potatoes [8] | Gilt-head bream [3] |
| *P.*  *trivialis* | Pork |  |  | Chicken [6] | Grasses [9] | |
| *P.*  *veronii* |  | Leafy  greens | |  | Turfgrass [10] | |

***Pseudomonas* phylogenetic trees**

Phylogenetic trees were created for *Pseudomonas* species where we had more than two genomes: *P. aeruginosa*, *P. fluorescens*, *P. fragi*, *P. koreensis* and *P. putida*. There were no multi-locus sequence typing schemes available for *P. fluorescens,* *P. fragi*, *P. koreensis* or *P. putida*. Instead clusters were predicted using TreeCluster [11].

The 51 *P. fluorescens* genomes isolated from food samples were compared to 33 publicly available *P. fluorescens* genomes (Figure S1). One of the food *P. fluorescens* genomes was in the same clade as genomes from plants and the environment, and another was in the same clade as a genome from a food sample from another study, but the remaining food isolates from this study were either within clades that only included food samples from this study or were within clades that only consisted of that individual isolate (singletons). Of the 25 food samples from which *P. fluorescens* was isolated, we had multiple *P. fluorescens* genomes from 16 samples and multiple *P. fluorescens* genomes belonging to different clades for 10 of these 16 samples.

**
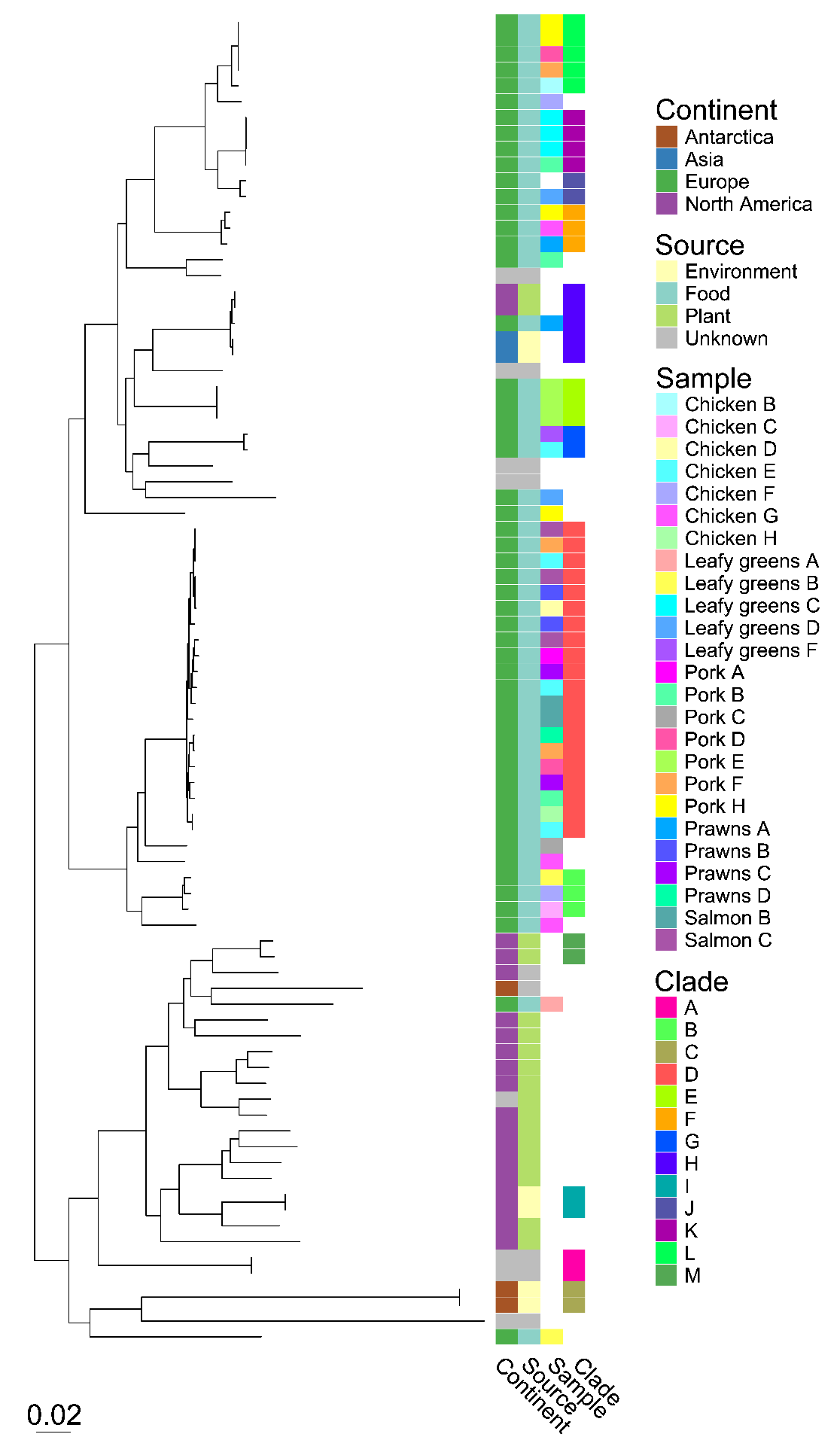
**

**Figure S1**. Maximum likelihood tree of 84 *P. fluorescens* isolates, colored by continent, source, sample and clade. The phylogenetic branch lengths are given in nucleotide substitutions per site, therefore a branch of length 0.02 (as represented by the scale bar) equates to 12,370 substitutions, given that the core gene alignment consisted of 618,488 bp.

No external *P. fragi* genomes were identified, so the 17 *P. fragi* genomes from food isolated in this study were compared with each other (Figure S2). Of the 11 food samples from which *P. fragi* was isolated, we had multiple *P. fragi* genomes from four samples and multiple *P. fragi* genomes belonging to different clades for two of these four samples.

**
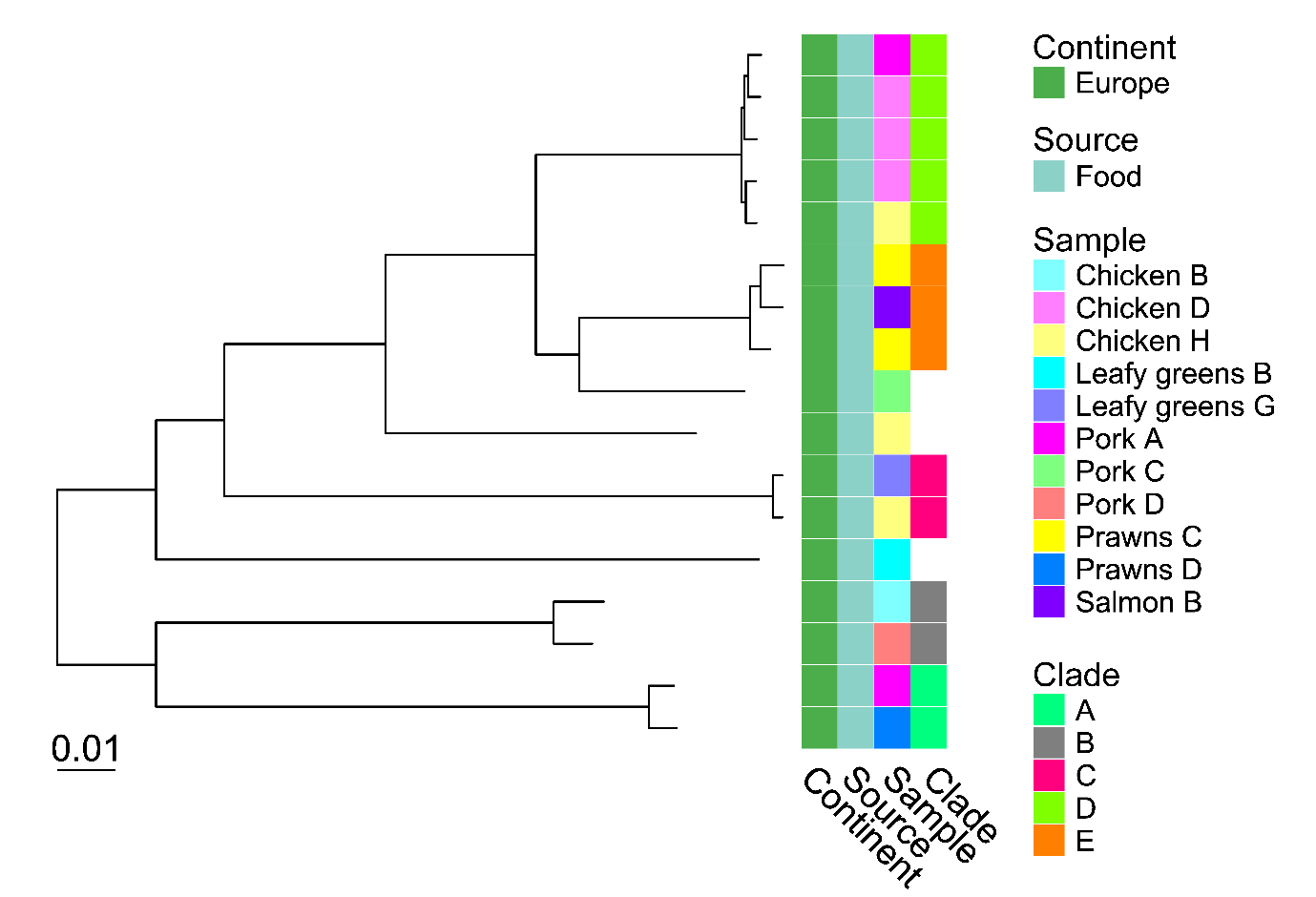
**

**Figure S2**. Maximum likelihood tree of 17 *P. fragi* isolates, colored by continent, source, sample and clade. The phylogenetic branch lengths are given in nucleotide substitutions per site, therefore a branch of length 0.01 (as represented by the scale bar) equates to 9,387 substitutions, given that the core gene alignment consisted of 938,722 bp.

The five *P. koreensis* genomes isolated from food were compared to 10 publicly available *P. koreensis* genomes (Figure S3). Three of the food *P. koreensis* genomes were in the same clade as a genome from a food sample from another study, and the other two were singletons. Of the four food samples from which *P. koreensis* was isolated, we had multiple *P. koreensis* genomes from one sample, but the genomes from this sample belonged to the same clade.

**
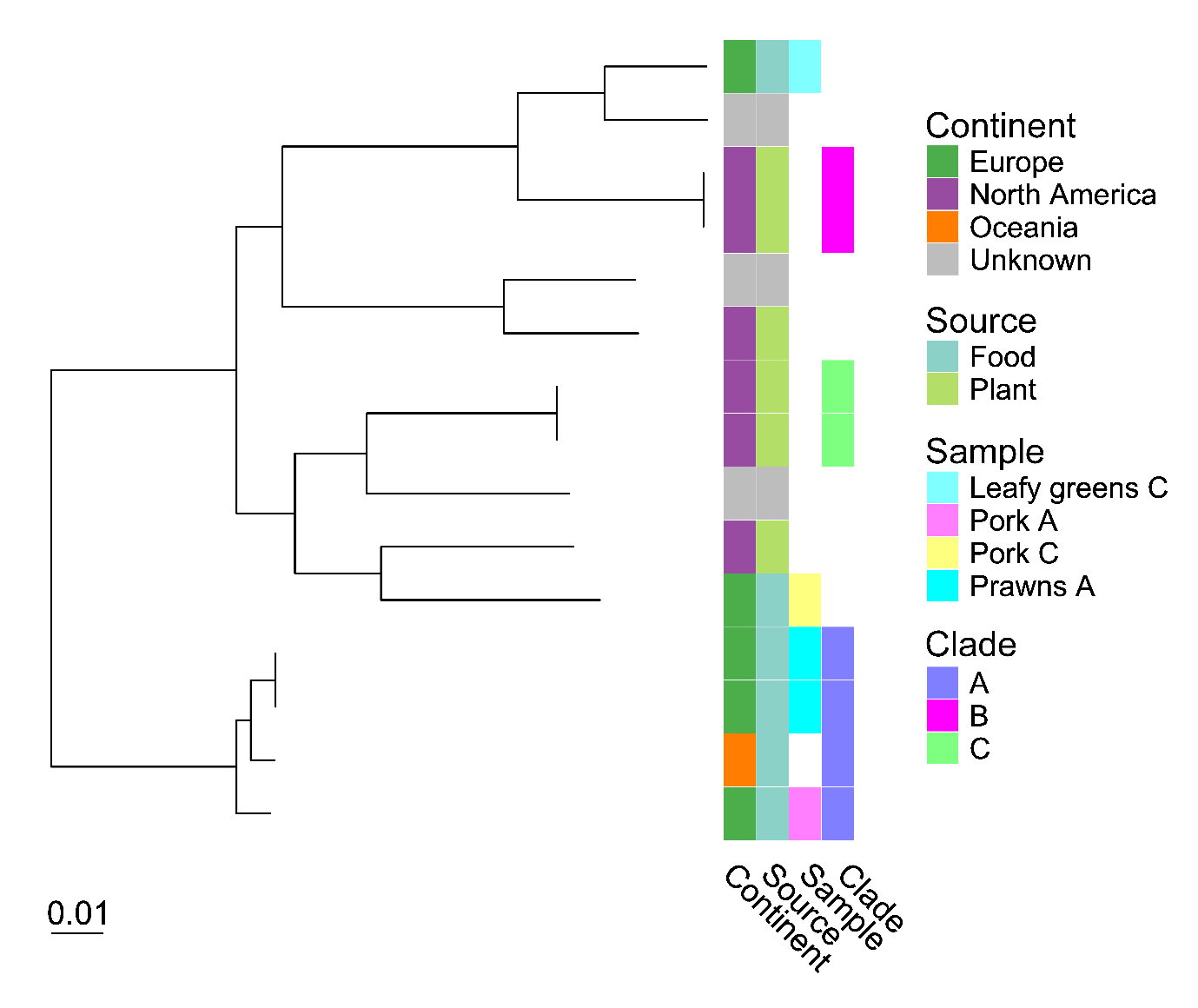
**

**Figure S3**. Maximum likelihood tree of 15 *P. koreensis* isolates, colored by continent, source, sample and clade. The phylogenetic branch lengths are given in nucleotide substitutions per site, therefore a branch of length 0.01 (as represented by the scale bar) equates to 21,723 substitutions, given that the core gene alignment consisted of 2,172,299 bp.

The 17 *P. putida* genomes isolated from food were compared to 17 *P. putida* publicly available genomes (Figure S4). Two of the food *P. putida* genomes were in the same clade as an external genome of unknown origin, whilst the rest were either within clades that only included food samples from this study or were singletons. Of the nine food samples from which *P. putida* was isolated, we had multiple *P. putida* genomes from five samples and multiple *P. putida* genomes belonging to different clades for three of these five samples.

**
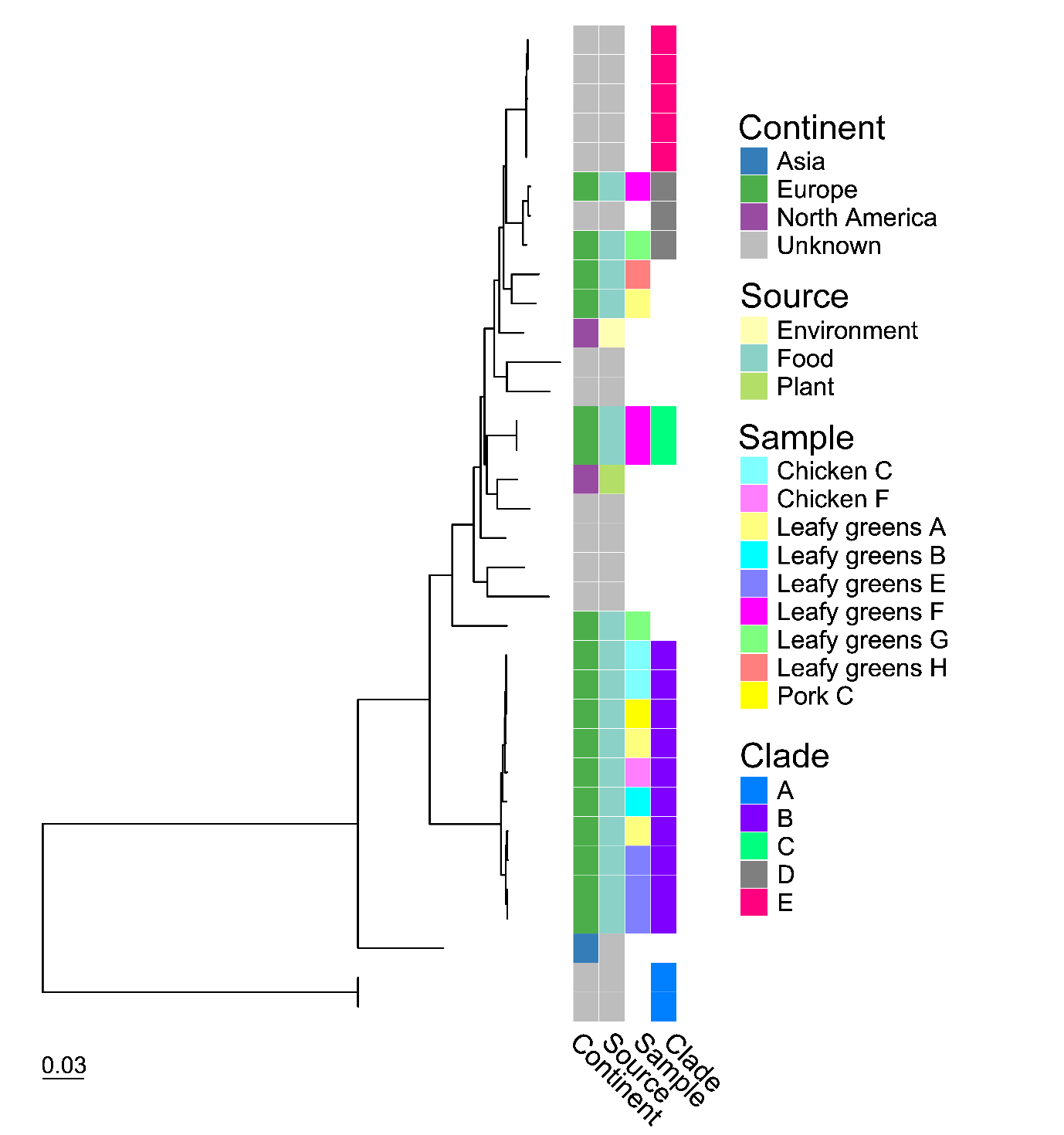
**

**Figure S4**. Maximum likelihood tree of 34 *P. putida* isolates, colored by continent, source, sample and clade. The phylogenetic branch lengths are given in nucleotide substitutions per site, therefore a branch of length 0.03 (as represented by the scale bar) equates to 227 substitutions, given that the core gene alignment consisted of 7,575 bp.

| **Table S3**. Metadata of *Pseudomonas aeruginosa* isolates cultured from food. | | | | | |
| --- | --- | --- | --- | --- | --- |
| Isolate | Sample | Species | Commodity | Wash status | Accession |
| PS21CH-0312-1 | CH-0312 | *P. aeruginosa* | Chicken | NA | SRR24625974 |
| PS21CH-0312-2 | CH-0312 | *P. aeruginosa* | Chicken | NA | SRR24625973 |
| PS21CH-0312-3 | CH-0312 | *P. aeruginosa* | Chicken | NA | SRR24625972 |
| PS21CH-0312-4 | CH-0312 | *P. aeruginosa* | Chicken | NA | SRR24625971 |
| PS21CH-0317-1 | CH-0317 | *P. aeruginosa* | Chicken | NA | SRR24625970 |
| PS21CH-0317-2 | CH-0317 | *P. aeruginosa* | Chicken | NA | SRR24625969 |
| PS21CH-0317-3 | CH-0317 | *P. aeruginosa* | Chicken | NA | SRR24625968 |
| PS21CH-0317-4 | CH-0317 | *P. aeruginosa* | Chicken | NA | SRR24626081 |
| PS21CH-0318-1 | CH-0318 | *P. aeruginosa* | Chicken | NA | SRR24626080 |
| PS21CH-0318-2 | CH-0318 | *P. aeruginosa* | Chicken | NA | SRR24626079 |
| PS21CH-0318-3 | CH-0318 | *P. aeruginosa* | Chicken | NA | SRR24626078 |
| PS21CH-0318-4 | CH-0318 | *P. aeruginosa* | Chicken | NA | SRR24626077 |
| PS21CH-0326-1 | CH-0326 | *P. aeruginosa* | Chicken | NA | SRR24626076 |
| PS21CH-0326-2 | CH-0326 | *P. aeruginosa* | Chicken | NA | SRR24626075 |
| PS21CH-0326-3 | CH-0326 | *P. aeruginosa* | Chicken | NA | SRR24626074 |
| PS21CH-0326-4 | CH-0326 | *P. aeruginosa* | Chicken | NA | SRR24626073 |
| PS21CH-0337-1 | CH-0337 | *P. aeruginosa* | Chicken | NA | SRR24626072 |
| PS21CH-0337-2 | CH-0337 | *P. aeruginosa* | Chicken | NA | SRR24626070 |
| PS21CH-0337-3 | CH-0337 | *P. aeruginosa* | Chicken | NA | SRR24626069 |
| PS21CH-0337-4 | CH-0337 | *P. aeruginosa* | Chicken | NA | SRR24626068 |
| PS21CH-0342-1 | CH-0342 | *P. aeruginosa* | Chicken | NA | SRR24626067 |
| PS21CH-0342-2 | CH-0342 | *P. aeruginosa* | Chicken | NA | SRR24626066 |
| PS21CH-0342-3 | CH-0342 | *P. aeruginosa* | Chicken | NA | SRR24626065 |
| PS21CH-0342-4 | CH-0342 | *P. aeruginosa* | Chicken | NA | SRR24626064 |
| PS21CH-0350-1 | CH-0350 | *P. aeruginosa* | Chicken | NA | SRR24626063 |
| PS21CH-0350-2 | CH-0350 | *P. aeruginosa* | Chicken | NA | SRR24626062 |
| PS21CH-0350-3 | CH-0350 | *P. aeruginosa* | Chicken | NA | SRR24626061 |
| PS21CH-0350-4 | CH-0350 | *P. aeruginosa* | Chicken | NA | SRR24626059 |
| PS21SM-0161-1 | SM-0161 | *P. aeruginosa* | Salmon | NA | SRR24626032 |
| PS21SM-0161-2 | SM-0161 | *P. aeruginosa* | Salmon | NA | SRR24626031 |
| PS21SM-0161-3 | SM-0161 | *P. aeruginosa* | Salmon | NA | SRR24626030 |
| PS21SM-0161-4 | SM-0161 | *P. aeruginosa* | Salmon | NA | SRR24626029 |
| PS22LB-0013-1 | LB-0013 | *P. aeruginosa* | Lamb | NA | SRR24626056 |
| PS22LB-0013-2 | LB-0013 | *P. aeruginosa* | Lamb | NA | SRR24626058 |
| PS22LB-0013-3 | LB-0013 | *P. aeruginosa* | Lamb | NA | SRR24626057 |
| PS22LB-0013-4 | LB-0013 | *P. aeruginosa* | Lamb | NA | SRR24626055 |
| PS22LG-0327-1 | LG-0327 | *P. aeruginosa* | Leafy greens | Washed | SRR24626054 |
| PS22LG-0327-2 | LG-0327 | *P. aeruginosa* | Leafy greens | Washed | SRR24626053 |
| PS22LG-0327-3 | LG-0327 | *P. aeruginosa* | Leafy greens | Washed | SRR24626052 |
| PS22LG-0327-4 | LG-0327 | *P. aeruginosa* | Leafy greens | Washed | SRR24626051 |
| PS22LG-0328-1 | LG-0328 | *P. aeruginosa* | Leafy greens | Washed | SRR24626050 |
| PS22LG-0328-2 | LG-0328 | *P. aeruginosa* | Leafy greens | Washed | SRR24626048 |
| PS22LG-0328-3 | LG-0328 | *P. aeruginosa* | Leafy greens | Washed | SRR24626047 |
| PS22LG-0328-4 | LG-0328 | *P. aeruginosa* | Leafy greens | Washed | SRR24626046 |
| PS22LG-0329-1 | LG-0329 | *P. aeruginosa* | Leafy greens | Washed | SRR24626045 |
| PS22LG-0329-2 | LG-0329 | *P. aeruginosa* | Leafy greens | Washed | SRR24626044 |
| PS22LG-0329-3 | LG-0329 | *P. aeruginosa* | Leafy greens | Washed | SRR24626043 |
| PS22LG-0329-4 | LG-0329 | *P. aeruginosa* | Leafy greens | Washed | SRR24626042 |
| PS22LG-0334-1 | LG-0334 | *P. aeruginosa* | Leafy greens | Washed | SRR24626041 |
| PS22LG-0334-2 | LG-0334 | *P. aeruginosa* | Leafy greens | Washed | SRR24626037 |
| PS22LG-0334-3 | LG-0334 | *P. aeruginosa* | Leafy greens | Washed | SRR24626040 |
| PS22LG-0334-4 | LG-0334 | *P. aeruginosa* | Leafy greens | Washed | SRR24626039 |
| PS22LG-0335-1 | LG-0335 | *P. aeruginosa* | Leafy greens | Washed | SRR24626036 |
| PS22LG-0335-2 | LG-0335 | *P. aeruginosa* | Leafy greens | Washed | SRR24626035 |
| PS22LG-0335-3 | LG-0335 | *P. aeruginosa* | Leafy greens | Washed | SRR24626034 |
| PS22LG-0335-4 | LG-0335 | *P. aeruginosa* | Leafy greens | Washed | SRR24626033 |

**Table S4.** *P. aeruginosa* sequence types and allele profiles responsible.

| ST | acsA | aroE | guaA | mutL | nuoD | ppsA | trpE |
| --- | --- | --- | --- | --- | --- | --- | --- |
| 9 | 6 | 6 | 4 | 3 | 3 | 4 | 7 |
| 17 | 11 | 5 | 1 | 7 | 9 | 4 | 7 |
| 27 | 6 | 5 | 6 | 7 | 4 | 6 | 7 |
| 111 | 17 | 5 | 5 | 4 | 4 | 4 | 3 |
| 115 | 28 | 5 | 36 | 11 | 4 | 42 | 7 |
| 132 | 6 | 20 | 1 | 3 | 4 | 4 | 2 |
| 146 | 6 | 5 | 11 | 3 | 4 | 23 | 1 |
| 152 | 6 | 5 | 19 | 3 | 4 | 6 | 7 |
| 155 | 28 | 5 | 36 | 3 | 3 | 13 | 7 |
| 162 | 6 | 5 | 6 | 34 | 27 | 3 | 7 |
| 175 | 28 | 22 | 5 | 3 | 3 | 14 | 19 |
| 179 | 36 | 27 | 28 | 3 | 4 | 13 | 7 |
| 198 | 11 | 5 | 11 | 11 | 3 | 27 | 7 |
| 233 | 16 | 5 | 30 | 11 | 4 | 31 | 41 |
| 235 | 38 | 11 | 3 | 13 | 1 | 2 | 4 |
| 236 | 28 | 5 | 1 | 5 | 4 | 32 | 10 |
| 244 | 17 | 5 | 12 | 3 | 14 | 4 | 7 |
| 252 | 6 | 28 | 4 | 3 | 3 | 4 | 7 |
| 253 | 4 | 4 | 16 | 12 | 1 | 6 | 3 |
| 260 | 14 | 5 | 10 | 7 | 4 | 13 | 7 |
| 261 | 105 | 5 | 30 | 3 | 3 | 4 | 14 |
| 262 | 17 | 5 | 1 | 3 | 4 | 15 | 7 |
| 267 | 19 | 5 | 12 | 11 | 11 | 4 | 14 |
| 274 | 23 | 5 | 11 | 7 | 1 | 12 | 7 |
| 277 | 39 | 5 | 9 | 11 | 27 | 5 | 2 |
| 279 | 5 | 3 | 57 | 3 | 1 | 33 | 47 |
| 282 | 6 | 5 | 11 | 7 | 3 | 12 | 19 |
| 291 | 6 | 5 | 5 | 3 | 52 | 4 | 2 |
| 298 | 18 | 4 | 13 | 3 | 1 | 17 | 13 |
| 308 | 13 | 4 | 5 | 5 | 12 | 7 | 15 |
| 313 | 47 | 8 | 7 | 6 | 8 | 11 | 40 |
| 316 | 13 | 8 | 9 | 3 | 1 | 6 | 9 |
| 319 | 5 | 4 | 3 | 3 | 1 | 11 | 8 |
| 347 | 40 | 5 | 17 | 5 | 4 | 15 | 7 |
| 348 | 22 | 20 | 11 | 3 | 3 | 3 | 7 |
| 357 | 2 | 4 | 5 | 3 | 1 | 6 | 11 |
| 360 | 15 | 5 | 36 | 11 | 27 | 4 | 2 |
| 377 | 103 | 8 | 5 | 5 | 1 | 6 | 4 |
| 381 | 11 | 20 | 1 | 65 | 4 | 4 | 10 |
| 385 | 15 | 5 | 5 | 5 | 50 | 4 | 14 |
| 386 | 17 | 5 | 11 | 18 | 4 | 10 | 3 |
| 388 | 17 | 14 | 11 | 11 | 1 | 15 | 2 |
| 389 | 17 | 22 | 5 | 3 | 1 | 14 | 3 |
| 390 | 39 | 5 | 1 | 3 | 4 | 46 | 56 |
| 395 | 6 | 5 | 1 | 1 | 1 | 12 | 1 |
| 412 | 11 | 8 | 11 | 5 | 4 | 4 | 7 |
| 439 | 6 | 68 | 20 | 11 | 4 | 4 | 7 |
| 443 | 15 | 5 | 5 | 5 | 50 | 4 | 1 |
| 446 | 18 | 4 | 5 | 3 | 1 | 17 | 13 |
| 463 | 6 | 5 | 5 | 3 | 1 | 6 | 3 |
| 498 | 6 | 28 | 65 | 3 | 3 | 4 | 7 |
| 500 | 11 | 57 | 7 | 3 | 4 | 15 | 1 |
| 508 | 15 | 5 | 11 | 3 | 2 | 4 | 3 |
| 532 | 5 | 4 | 5 | 5 | 5 | 20 | 4 |
| 549 | 7 | 5 | 12 | 3 | 4 | 1 | 7 |
| 553 | 17 | 5 | 1 | 11 | 4 | 4 | 45 |
| 558 | 40 | 22 | 1 | 3 | 2 | 6 | 7 |
| 569 | 11 | 5 | 11 | 11 | 3 | 6 | 27 |
| 612 | 28 | 5 | 58 | 11 | 4 | 15 | 44 |
| 620 | 9 | 7 | 63 | 13 | 8 | 7 | 8 |
| 621 | 15 | 5 | 20 | 5 | 1 | 4 | 25 |
| 640 | 11 | 5 | 98 | 5 | 3 | 10 | 85 |
| 641 | 6 | 5 | 6 | 5 | 4 | 4 | 7 |
| 645 | 6 | 5 | 5 | 3 | 3 | 13 | 1 |
| 646 | 11 | 5 | 6 | 11 | 2 | 4 | 19 |
| 654 | 17 | 5 | 26 | 3 | 4 | 4 | 26 |
| 664 | 9 | 5 | 11 | 3 | 4 | 40 | 18 |
| 667 | 5 | 67 | 76 | 3 | 1 | 7 | 62 |
| 671 | 5 | 5 | 57 | 13 | 1 | 74 | 3 |
| 675 | 40 | 84 | 11 | 3 | 4 | 76 | 91 |
| 676 | 28 | 5 | 11 | 77 | 3 | 4 | 92 |
| 677 | 28 | 5 | 36 | 102 | 3 | 13 | 7 |
| 699 | 7 | 5 | 7 | 3 | 4 | 1 | 7 |
| 712 | 31 | 12 | 65 | 19 | 13 | 7 | 23 |
| 782 | 15 | 3 | 3 | 11 | 1 | 15 | 1 |
| 792 | 6 | 5 | 11 | 3 | 2 | 15 | 1 |
| 794 | 17 | 22 | 5 | 11 | 3 | 86 | 19 |
| 796 | 36 | 5 | 1 | 3 | 2 | 6 | 1 |
| 800 | 17 | 22 | 11 | 3 | 3 | 15 | 3 |
| 815 | 103 | 11 | 61 | 5 | 1 | 6 | 8 |
| 830 | 5 | 13 | 109 | 5 | 1 | 1 | 47 |
| 845 | 11 | 5 | 1 | 7 | 4 | 4 | 7 |
| 871 | 16 | 3 | 1 | 5 | 1 | 55 | 61 |
| 901 | 17 | 5 | 12 | 11 | 4 | 4 | 68 |
| 926 | 29 | 1 | 97 | 99 | 24 | 20 | 87 |
| 959 | 6 | 5 | 11 | 7 | 3 | 70 | 19 |
| 964 | 145 | 5 | 26 | 3 | 4 | 4 | 26 |
| 966 | 17 | 3 | 5 | 4 | 4 | 4 | 3 |
| 1025 | 6 | 5 | 5 | 3 | 3 | 13 | 26 |
| 1076 | 5 | 4 | 57 | 62 | 1 | 1 | 26 |
| 1101 | 16 | 134 | 11 | 7 | 4 | 15 | 19 |
| 1105 | 23 | 5 | 12 | 30 | 1 | 4 | 7 |
| 1125 | 11 | 5 | 11 | 13 | 3 | 4 | 1 |
| 1129 | 22 | 5 | 91 | 3 | 4 | 4 | 7 |
| 1144 | 39 | 10 | 1 | 3 | 4 | 6 | 7 |
| 1194 | 28 | 24 | 10 | 5 | 1 | 6 | 2 |
| 1203 | 33 | 1 | 25 | 6 | 6 | 7 | 5 |
| 1212 | 11 | 10 | 11 | 72 | 3 | 10 | 3 |
| 1239 | 16 | 5 | 1 | 3 | 4 | 15 | 7 |
| 1247 | 15 | 5 | 77 | 72 | 3 | 6 | 68 |
| 1275 | 28 | 107 | 1 | 3 | 1 | 6 | 45 |
| 1320 | 22 | 20 | 122 | 3 | 3 | 3 | 7 |
| 1342 | 1 | 5 | 26 | 3 | 1 | 10 | 3 |
| 1395 | 25 | 5 | 36 | 5 | 4 | 117 | 7 |
| 1399 | 28 | 10 | 1 | 3 | 27 | 4 | 7 |
| 1455 | 15 | 5 | 11 | 3 | 58 | 42 | 9 |
| 1527 | 17 | 10 | 129 | 5 | 4 | 112 | 19 |
| 1567 | 32 | 8 | 3 | 18 | 1 | 123 | 118 |
| 1591 | 16 | 5 | 11 | 11 | 4 | 13 | 7 |
| 1600 | 16 | 5 | 19 | 3 | 4 | 13 | 7 |
| 1610 | 17 | 4 | 16 | 12 | 1 | 6 | 3 |
| 1621 | 5 | 54 | 99 | 48 | 1 | 6 | 3 |
| 1664 | 17 | 18 | 17 | 5 | 4 | 4 | 21 |
| 1682 | 39 | 6 | 4 | 14 | 4 | 15 | 2 |
| 1693 | 64 | 5 | 11 | 5 | 2 | 4 | 19 |
| 1800 | 11 | 5 | 73 | 3 | 4 | 4 | 3 |
| 1990 | 5 | 14 | 25 | 5 | 16 | 7 | 28 |
| 2021 | 11 | 5 | 37 | 34 | 4 | 13 | 7 |
| 2022 | 16 | 76 | 7 | 11 | 44 | 6 | 45 |
| 2024 | 23 | 5 | 11 | 7 | 4 | 12 | 7 |
| 2025 | 11 | 196 | 12 | 34 | 4 | 13 | 18 |
| 2027 | 17 | 3 | 11 | 11 | 4 | 38 | 7 |
| 2029 | 15 | 28 | 11 | 3 | 3 | 38 | 10 |
| 2030 | 5 | 61 | 79 | 11 | 10 | 7 | 124 |
| 2032 | 5 | 8 | 115 | 28 | 10 | 7 | 5 |
| 2033 | 15 | 5 | 30 | 72 | 3 | 6 | 68 |
| 2034 | 40 | 5 | 142 | 162 | 73 | 75 | 60 |
| 2035 | 16 | 14 | 3 | 11 | 1 | 132 | 1 |
| 2037 | 15 | 199 | 5 | 5 | 50 | 4 | 14 |
| 2038 | 5 | 200 | 11 | 6 | 1 | 101 | 139 |
| 2040 | 47 | 8 | 7 | 163 | 8 | 11 | 40 |
| 2041 | 16 | 22 | 19 | 11 | 3 | 15 | 2 |
| 2044 | 40 | 5 | 17 | 3 | 4 | 15 | 2 |
| 2045 | 6 | 5 | 4 | 11 | 2 | 6 | 25 |
| 2046 | 5 | 4 | 143 | 5 | 5 | 20 | 4 |
| 2048 | 40 | 5 | 11 | 5 | 2 | 4 | 37 |
| 2049 | 32 | 4 | 5 | 5 | 1 | 36 | 26 |
| 2051 | 11 | 5 | 5 | 3 | 4 | 133 | 7 |
| 2053 | 16 | 5 | 4 | 74 | 2 | 7 | 10 |
| 2054 | 15 | 5 | 30 | 72 | 64 | 6 | 68 |
| 2056 | 11 | 122 | 36 | 164 | 3 | 12 | 27 |
| 2057 | 142 | 12 | 65 | 165 | 1 | 16 | 198 |
| 2058 | 16 | 5 | 11 | 11 | 2 | 15 | 1 |
| 2059 | 1 | 3 | 12 | 3 | 1 | 135 | 4 |
| 2060 | 40 | 202 | 145 | 7 | 1 | 12 | 1 |
| 2061 | 15 | 5 | 5 | 3 | 3 | 15 | 3 |
| 2123 | 6 | 5 | 36 | 3 | 3 | 15 | 2 |
| 2184 | 28 | 23 | 5 | 61 | 4 | 1 | 11 |
| 2699 | 11 | 10 | 6 | 5 | 27 | 6 | 9 |
| 2728 | 11 | 5 | 3 | 3 | 8 | 20 | 9 |
| 2729 | 5 | 141 | 65 | 151 | 1 | 33 | 50 |
| 2730 | 15 | 48 | 20 | 142 | 4 | 7 | 7 |
| 2733 | 40 | 5 | 3 | 162 | 73 | 75 | 2 |
| 3090 | 40 | 5 | 36 | 153 | 3 | 7 | 19 |
| 3373 | 5 | 208 | 60 | 10 | 1 | 2 | 8 |
| 3700 | 5 | 5 | 20 | 18 | 3 | 15 | 19 |
| Novel_A | 17 | 5 | 11 | 3 | 3 | 7 | 2 |
| Novel_B | 207 | 5 | 1 | 4 | 4 | 4 | 17 |
| Novel_C | 105 | 5 | 30 | 3 | 1 | 4 | 14 |
| Novel_D | 14 | 5 | 96 | 7 | 4 | 13 | 7 |
| Novel_E | 11 | 5 | 1 | 7 | 9 | 4 | 152 |
| Novel_F | 6 | 5 | 6 | 7 | 4 | 6 | 82 |
| Novel_G | 28 | 22 | 163 | 180 | 4 | 4 | 7 |
| Novel_H | 6 | 28 | 65 | 3 | 3 | 4 | 152 |
| Novel_I | 15 | 220 | 36 | 11 | 64 | 13 | 221 |
| Novel_J | 28 | 5 | 1 | 4 | 4 | 4 | 1 |
| Novel_K | 16 | 5 | 58 | 34 | 1 | 47 | 1 |
| Novel_L | 38 | 11 | 59 | 13 | 1 | 2 | 4 |
| Novel_M | 41 | 5 | 7 | 61 | 2 | 4 | 7 |

**
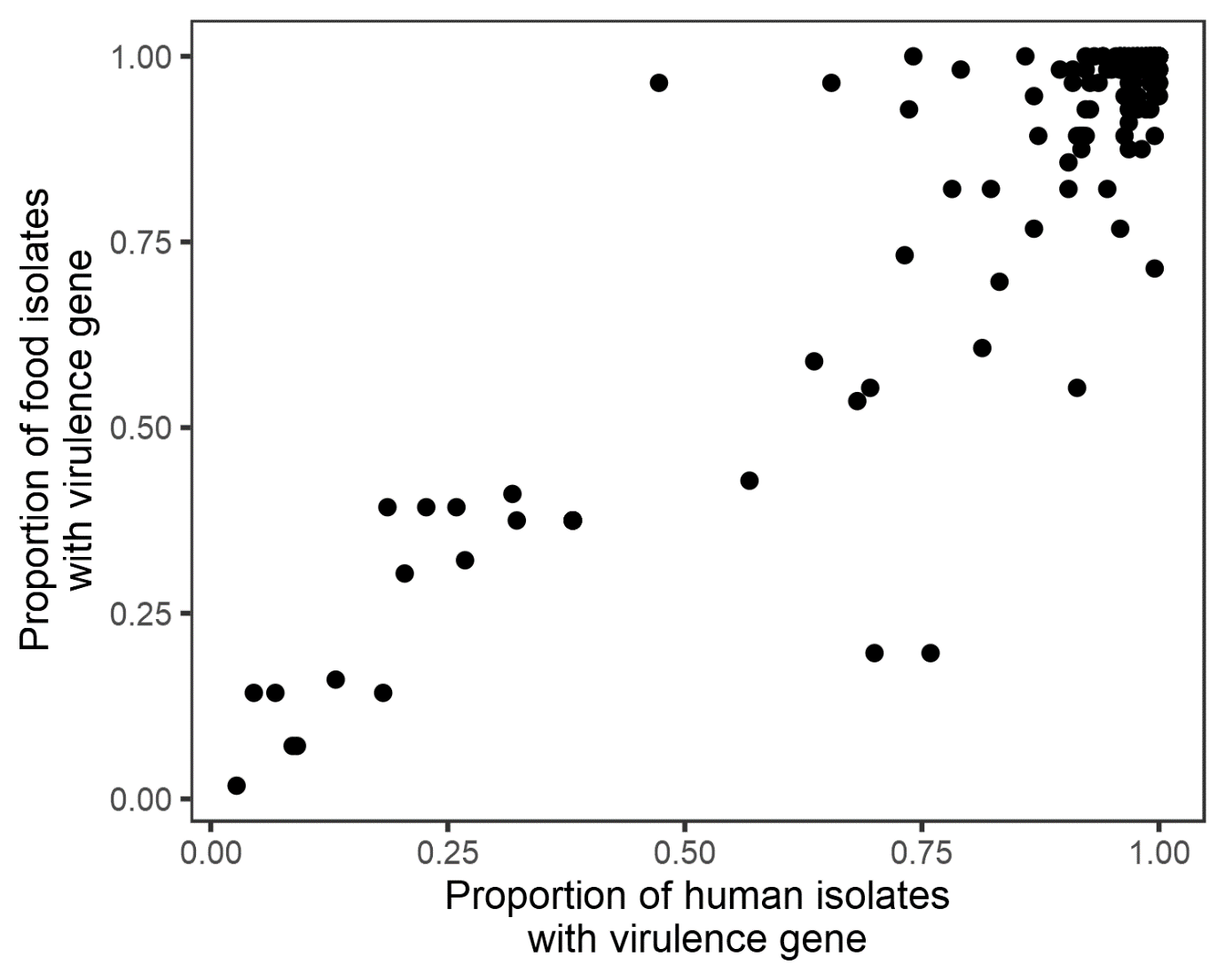
**

**Figure S5.** Proportion of *P. aeruginosa* isolates from human sources that contained virulence genes compared to those from food sources.

**Table S5.** Virulence genes that significantly differed amongst *P. aeruginosa* isolates collected from human and food sources.

| Gene | Food isolate proportion | Human isolate proportion | p-value |
| --- | --- | --- | --- |
| *mucA* | 1.00 | 0.741 | 2.63 x 10^-5^ |
| *pchE* | 0.196 | 0.759 | 9.08 x 10^-14^ |
| *pchG* | 0.768 | 0.959 | 3.81 x 10^-6^ |
| *pchH* | 0.554 | 0.914 | 2.50 x 10^-10^ |
| *ppkA* | 0.196 | 0.700 | 4.54 x 10^-11^ |
| *pscH* | 0.714 | 0.996 | 1.19 x 10^-13^ |
| *pvdM* | 0.964 | 0.473 | 1.69 x 10^-10^ |
| *pvdN* | 0.964 | 0.655 | 6.58 x 10^-6^ |
| *tagF/pppB* | 0.893 | 0.996 | 1.84 x 10^-5^ |

**Table S6.** Core single nucleotide polymorphism (SNP), antimicrobial resistance genes (ARGs) and virulence gene differences between food isolates and the closest-related human isolate.

|  |  |  | Differences | |  |  | Human isolate | |
| --- | --- | --- | --- | --- | --- | --- | --- | --- |
| Sample | Type | ST | SNPs | AMR | Plasmid | Virulence | Year | Country |
| CH-0342 | Chicken | 699 | 185 | 1 | 0 | 6 | 1991 | France |
| LB-0013 | Lamb | 319 | 312 | 2 | 0 | 7 | 1997 | Turkey |
| LG-0327 | Leafy greens | 253 | 41 | 0 | 0 | 3 | 1993 | France |
| SM-0161 | Salmon | 17 | 58 | 0 | 0 | 6 | Unknown | Unknown |

**Table S7.** *Galleria mellonella* larvae survival with different *P. aeruginosa* strains.

| Time (hours) | Survival (larvae alive) | Replicate | Strain | Proportion alive |
| --- | --- | --- | --- | --- |
| 0 | 10 | 1 | CH-0312-2 | 1 |
| 17 | 4 | 1 | CH-0312-2 | 0.4 |
| 20 | 4 | 1 | CH-0312-2 | 0.4 |
| 24 | 3 | 1 | CH-0312-2 | 0.3 |
| 41 | 3 | 1 | CH-0312-2 | 0.3 |
| 44 | 3 | 1 | CH-0312-2 | 0.3 |
| 49 | 3 | 1 | CH-0312-2 | 0.3 |
| 0 | 10 | 2 | CH-0312-2 | 1 |
| 17 | 0 | 2 | CH-0312-2 | 0 |
| 20 | 0 | 2 | CH-0312-2 | 0 |
| 24 | 0 | 2 | CH-0312-2 | 0 |
| 41 | 0 | 2 | CH-0312-2 | 0 |
| 44 | 0 | 2 | CH-0312-2 | 0 |
| 48 | 0 | 2 | CH-0312-2 | 0 |
| 0 | 10 | 3 | CH-0312-2 | 1 |
| 5 | 10 | 3 | CH-0312-2 | 1 |
| 21 | 10 | 3 | CH-0312-2 | 1 |
| 25 | 10 | 3 | CH-0312-2 | 1 |
| 29 | 10 | 3 | CH-0312-2 | 1 |
| 45 | 9 | 3 | CH-0312-2 | 0.9 |
| 49 | 9 | 3 | CH-0312-2 | 0.9 |
| 53 | 9 | 3 | CH-0312-2 | 0.9 |
| 0 | 10 | 1 | CH-0317-3 | 1 |
| 17 | 4 | 1 | CH-0317-3 | 0.4 |
| 20 | 3 | 1 | CH-0317-3 | 0.3 |
| 24 | 1 | 1 | CH-0317-3 | 0.1 |
| 41 | 1 | 1 | CH-0317-3 | 0.1 |
| 44 | 1 | 1 | CH-0317-3 | 0.1 |
| 49 | 0 | 1 | CH-0317-3 | 0 |
| 0 | 10 | 2 | CH-0317-3 | 1 |
| 17 | 4 | 2 | CH-0317-3 | 0.4 |
| 20 | 4 | 2 | CH-0317-3 | 0.4 |
| 24 | 2 | 2 | CH-0317-3 | 0.2 |
| 41 | 2 | 2 | CH-0317-3 | 0.2 |
| 44 | 2 | 2 | CH-0317-3 | 0.2 |
| 48 | 2 | 2 | CH-0317-3 | 0.2 |
| 0 | 10 | 3 | CH-0317-3 | 1 |
| 5 | 10 | 3 | CH-0317-3 | 1 |
| 21 | 8 | 3 | CH-0317-3 | 0.8 |
| 25 | 7 | 3 | CH-0317-3 | 0.7 |
| 29 | 7 | 3 | CH-0317-3 | 0.7 |
| 45 | 6 | 3 | CH-0317-3 | 0.6 |
| 49 | 6 | 3 | CH-0317-3 | 0.6 |
| 53 | 5 | 3 | CH-0317-3 | 0.5 |
| 0 | 10 | 1 | CH-0318-4 | 1 |
| 17 | 5 | 1 | CH-0318-4 | 0.5 |
| 20 | 3 | 1 | CH-0318-4 | 0.3 |
| 24 | 2 | 1 | CH-0318-4 | 0.2 |
| 41 | 2 | 1 | CH-0318-4 | 0.2 |
| 44 | 2 | 1 | CH-0318-4 | 0.2 |
| 49 | 2 | 1 | CH-0318-4 | 0.2 |
| 0 | 10 | 2 | CH-0318-4 | 1 |
| 17 | 1 | 2 | CH-0318-4 | 0.1 |
| 20 | 1 | 2 | CH-0318-4 | 0.1 |
| 24 | 1 | 2 | CH-0318-4 | 0.1 |
| 41 | 1 | 2 | CH-0318-4 | 0.1 |
| 44 | 1 | 2 | CH-0318-4 | 0.1 |
| 48 | 1 | 2 | CH-0318-4 | 0.1 |
| 0 | 10 | 3 | CH-0318-4 | 1 |
| 5 | 10 | 3 | CH-0318-4 | 1 |
| 21 | 5 | 3 | CH-0318-4 | 0.5 |
| 25 | 5 | 3 | CH-0318-4 | 0.5 |
| 29 | 5 | 3 | CH-0318-4 | 0.5 |
| 45 | 5 | 3 | CH-0318-4 | 0.5 |
| 49 | 5 | 3 | CH-0318-4 | 0.5 |
| 53 | 5 | 3 | CH-0318-4 | 0.5 |
| 0 | 10 | 1 | CH-0326-3 | 1 |
| 17 | 5 | 1 | CH-0326-3 | 0.5 |
| 20 | 3 | 1 | CH-0326-3 | 0.3 |
| 24 | 3 | 1 | CH-0326-3 | 0.3 |
| 41 | 3 | 1 | CH-0326-3 | 0.3 |
| 44 | 3 | 1 | CH-0326-3 | 0.3 |
| 49 | 3 | 1 | CH-0326-3 | 0.3 |
| 0 | 10 | 2 | CH-0326-3 | 1 |
| 17 | 0 | 2 | CH-0326-3 | 0 |
| 20 | 0 | 2 | CH-0326-3 | 0 |
| 24 | 0 | 2 | CH-0326-3 | 0 |
| 41 | 0 | 2 | CH-0326-3 | 0 |
| 44 | 0 | 2 | CH-0326-3 | 0 |
| 48 | 0 | 2 | CH-0326-3 | 0 |
| 0 | 10 | 3 | CH-0326-3 | 1 |
| 5 | 10 | 3 | CH-0326-3 | 1 |
| 21 | 7 | 3 | CH-0326-3 | 0.7 |
| 25 | 7 | 3 | CH-0326-3 | 0.7 |
| 29 | 7 | 3 | CH-0326-3 | 0.7 |
| 45 | 6 | 3 | CH-0326-3 | 0.6 |
| 49 | 6 | 3 | CH-0326-3 | 0.6 |
| 53 | 6 | 3 | CH-0326-3 | 0.6 |
| 0 | 10 | 1 | CH-0337-2 | 1 |
| 17 | 5 | 1 | CH-0337-2 | 0.5 |
| 20 | 3 | 1 | CH-0337-2 | 0.3 |
| 24 | 3 | 1 | CH-0337-2 | 0.3 |
| 41 | 3 | 1 | CH-0337-2 | 0.3 |
| 44 | 3 | 1 | CH-0337-2 | 0.3 |
| 49 | 3 | 1 | CH-0337-2 | 0.3 |
| 0 | 10 | 2 | CH-0337-2 | 1 |
| 17 | 0 | 2 | CH-0337-2 | 0 |
| 20 | 0 | 2 | CH-0337-2 | 0 |
| 24 | 0 | 2 | CH-0337-2 | 0 |
| 41 | 0 | 2 | CH-0337-2 | 0 |
| 44 | 0 | 2 | CH-0337-2 | 0 |
| 48 | 0 | 2 | CH-0337-2 | 0 |
| 0 | 10 | 3 | CH-0337-2 | 1 |
| 5 | 10 | 3 | CH-0337-2 | 1 |
| 21 | 7 | 3 | CH-0337-2 | 0.7 |
| 25 | 7 | 3 | CH-0337-2 | 0.7 |
| 29 | 7 | 3 | CH-0337-2 | 0.7 |
| 45 | 7 | 3 | CH-0337-2 | 0.7 |
| 49 | 7 | 3 | CH-0337-2 | 0.7 |
| 53 | 7 | 3 | CH-0337-2 | 0.7 |
| 0 | 10 | 1 | CH-0342-8 | 1 |
| 17 | 4 | 1 | CH-0342-8 | 0.4 |
| 20 | 1 | 1 | CH-0342-8 | 0.1 |
| 24 | 1 | 1 | CH-0342-8 | 0.1 |
| 41 | 1 | 1 | CH-0342-8 | 0.1 |
| 44 | 0 | 1 | CH-0342-8 | 0 |
| 49 | 0 | 1 | CH-0342-8 | 0 |
| 0 | 10 | 2 | CH-0342-8 | 1 |
| 17 | 3 | 2 | CH-0342-8 | 0.3 |
| 20 | 2 | 2 | CH-0342-8 | 0.2 |
| 24 | 2 | 2 | CH-0342-8 | 0.2 |
| 41 | 2 | 2 | CH-0342-8 | 0.2 |
| 44 | 2 | 2 | CH-0342-8 | 0.2 |
| 48 | 2 | 2 | CH-0342-8 | 0.2 |
| 0 | 10 | 3 | CH-0342-8 | 1 |
| 5 | 10 | 3 | CH-0342-8 | 1 |
| 21 | 6 | 3 | CH-0342-8 | 0.6 |
| 25 | 6 | 3 | CH-0342-8 | 0.6 |
| 29 | 6 | 3 | CH-0342-8 | 0.6 |
| 45 | 6 | 3 | CH-0342-8 | 0.6 |
| 49 | 6 | 3 | CH-0342-8 | 0.6 |
| 53 | 6 | 3 | CH-0342-8 | 0.6 |
| 0 | 10 | 1 | CH-0350-4 | 1 |
| 17 | 4 | 1 | CH-0350-4 | 0.4 |
| 20 | 1 | 1 | CH-0350-4 | 0.1 |
| 24 | 0 | 1 | CH-0350-4 | 0 |
| 41 | 0 | 1 | CH-0350-4 | 0 |
| 44 | 0 | 1 | CH-0350-4 | 0 |
| 49 | 0 | 1 | CH-0350-4 | 0 |
| 0 | 10 | 2 | CH-0350-4 | 1 |
| 17 | 2 | 2 | CH-0350-4 | 0.2 |
| 20 | 2 | 2 | CH-0350-4 | 0.2 |
| 24 | 2 | 2 | CH-0350-4 | 0.2 |
| 41 | 2 | 2 | CH-0350-4 | 0.2 |
| 44 | 2 | 2 | CH-0350-4 | 0.2 |
| 48 | 2 | 2 | CH-0350-4 | 0.2 |
| 0 | 10 | 3 | CH-0350-4 | 1 |
| 5 | 10 | 3 | CH-0350-4 | 1 |
| 21 | 6 | 3 | CH-0350-4 | 0.6 |
| 25 | 6 | 3 | CH-0350-4 | 0.6 |
| 29 | 6 | 3 | CH-0350-4 | 0.6 |
| 45 | 6 | 3 | CH-0350-4 | 0.6 |
| 49 | 5 | 3 | CH-0350-4 | 0.5 |
| 53 | 5 | 3 | CH-0350-4 | 0.5 |
| 0 | 10 | 1 | LB-0013-1 | 1 |
| 17 | 8 | 1 | LB-0013-1 | 0.8 |
| 20 | 3 | 1 | LB-0013-1 | 0.3 |
| 24 | 3 | 1 | LB-0013-1 | 0.3 |
| 41 | 3 | 1 | LB-0013-1 | 0.3 |
| 44 | 3 | 1 | LB-0013-1 | 0.3 |
| 49 | 3 | 1 | LB-0013-1 | 0.3 |
| 0 | 10 | 2 | LB-0013-1 | 1 |
| 17 | 0 | 2 | LB-0013-1 | 0 |
| 20 | 0 | 2 | LB-0013-1 | 0 |
| 24 | 0 | 2 | LB-0013-1 | 0 |
| 41 | 0 | 2 | LB-0013-1 | 0 |
| 44 | 0 | 2 | LB-0013-1 | 0 |
| 48 | 0 | 2 | LB-0013-1 | 0 |
| 0 | 10 | 3 | LB-0013-1 | 1 |
| 5 | 10 | 3 | LB-0013-1 | 1 |
| 21 | 9 | 3 | LB-0013-1 | 0.9 |
| 25 | 9 | 3 | LB-0013-1 | 0.9 |
| 29 | 9 | 3 | LB-0013-1 | 0.9 |
| 45 | 9 | 3 | LB-0013-1 | 0.9 |
| 49 | 9 | 3 | LB-0013-1 | 0.9 |
| 53 | 9 | 3 | LB-0013-1 | 0.9 |
| 0 | 10 | 1 | LB-0013-3 | 1 |
| 17 | 6 | 1 | LB-0013-3 | 0.6 |
| 20 | 3 | 1 | LB-0013-3 | 0.3 |
| 24 | 3 | 1 | LB-0013-3 | 0.3 |
| 41 | 3 | 1 | LB-0013-3 | 0.3 |
| 44 | 3 | 1 | LB-0013-3 | 0.3 |
| 49 | 3 | 1 | LB-0013-3 | 0.3 |
| 0 | 10 | 2 | LB-0013-3 | 1 |
| 17 | 0 | 2 | LB-0013-3 | 0 |
| 20 | 0 | 2 | LB-0013-3 | 0 |
| 24 | 0 | 2 | LB-0013-3 | 0 |
| 41 | 0 | 2 | LB-0013-3 | 0 |
| 44 | 0 | 2 | LB-0013-3 | 0 |
| 48 | 0 | 2 | LB-0013-3 | 0 |
| 0 | 10 | 3 | LB-0013-3 | 1 |
| 5 | 10 | 3 | LB-0013-3 | 1 |
| 21 | 8 | 3 | LB-0013-3 | 0.8 |
| 25 | 6 | 3 | LB-0013-3 | 0.6 |
| 29 | 6 | 3 | LB-0013-3 | 0.6 |
| 45 | 6 | 3 | LB-0013-3 | 0.6 |
| 49 | 6 | 3 | LB-0013-3 | 0.6 |
| 53 | 6 | 3 | LB-0013-3 | 0.6 |
| 0 | 10 | 1 | LG-0327-3 | 1 |
| 17 | 8 | 1 | LG-0327-3 | 0.8 |
| 20 | 7 | 1 | LG-0327-3 | 0.7 |
| 24 | 7 | 1 | LG-0327-3 | 0.7 |
| 41 | 7 | 1 | LG-0327-3 | 0.7 |
| 44 | 7 | 1 | LG-0327-3 | 0.7 |
| 49 | 7 | 1 | LG-0327-3 | 0.7 |
| 0 | 10 | 2 | LG-0327-3 | 1 |
| 17 | 6 | 2 | LG-0327-3 | 0.6 |
| 20 | 5 | 2 | LG-0327-3 | 0.5 |
| 24 | 4 | 2 | LG-0327-3 | 0.4 |
| 41 | 4 | 2 | LG-0327-3 | 0.4 |
| 44 | 4 | 2 | LG-0327-3 | 0.4 |
| 48 | 4 | 2 | LG-0327-3 | 0.4 |
| 0 | 10 | 3 | LG-0327-3 | 1 |
| 5 | 10 | 3 | LG-0327-3 | 1 |
| 21 | 6 | 3 | LG-0327-3 | 0.6 |
| 25 | 6 | 3 | LG-0327-3 | 0.6 |
| 29 | 6 | 3 | LG-0327-3 | 0.6 |
| 45 | 6 | 3 | LG-0327-3 | 0.6 |
| 49 | 6 | 3 | LG-0327-3 | 0.6 |
| 53 | 6 | 3 | LG-0327-3 | 0.6 |
| 0 | 10 | 1 | LG-0328-1 | 1 |
| 17 | 3 | 1 | LG-0328-1 | 0.3 |
| 20 | 1 | 1 | LG-0328-1 | 0.1 |
| 24 | 1 | 1 | LG-0328-1 | 0.1 |
| 41 | 1 | 1 | LG-0328-1 | 0.1 |
| 44 | 1 | 1 | LG-0328-1 | 0.1 |
| 49 | 0 | 1 | LG-0328-1 | 0 |
| 0 | 10 | 2 | LG-0328-1 | 1 |
| 17 | 4 | 2 | LG-0328-1 | 0.4 |
| 20 | 3 | 2 | LG-0328-1 | 0.3 |
| 24 | 3 | 2 | LG-0328-1 | 0.3 |
| 41 | 3 | 2 | LG-0328-1 | 0.3 |
| 44 | 3 | 2 | LG-0328-1 | 0.3 |
| 48 | 3 | 2 | LG-0328-1 | 0.3 |
| 0 | 10 | 3 | LG-0328-1 | 1 |
| 5 | 10 | 3 | LG-0328-1 | 1 |
| 21 | 8 | 3 | LG-0328-1 | 0.8 |
| 25 | 8 | 3 | LG-0328-1 | 0.8 |
| 29 | 8 | 3 | LG-0328-1 | 0.8 |
| 45 | 8 | 3 | LG-0328-1 | 0.8 |
| 49 | 8 | 3 | LG-0328-1 | 0.8 |
| 53 | 8 | 3 | LG-0328-1 | 0.8 |
| 0 | 10 | 1 | LG-0329-4 | 1 |
| 17 | 6 | 1 | LG-0329-4 | 0.6 |
| 20 | 2 | 1 | LG-0329-4 | 0.2 |
| 24 | 2 | 1 | LG-0329-4 | 0.2 |
| 41 | 2 | 1 | LG-0329-4 | 0.2 |
| 44 | 2 | 1 | LG-0329-4 | 0.2 |
| 49 | 2 | 1 | LG-0329-4 | 0.2 |
| 0 | 10 | 2 | LG-0329-4 | 1 |
| 17 | 2 | 2 | LG-0329-4 | 0.2 |
| 20 | 2 | 2 | LG-0329-4 | 0.2 |
| 24 | 1 | 2 | LG-0329-4 | 0.1 |
| 41 | 1 | 2 | LG-0329-4 | 0.1 |
| 44 | 1 | 2 | LG-0329-4 | 0.1 |
| 48 | 1 | 2 | LG-0329-4 | 0.1 |
| 0 | 10 | 3 | LG-0329-4 | 1 |
| 5 | 10 | 3 | LG-0329-4 | 1 |
| 21 | 5 | 3 | LG-0329-4 | 0.5 |
| 25 | 5 | 3 | LG-0329-4 | 0.5 |
| 29 | 5 | 3 | LG-0329-4 | 0.5 |
| 45 | 5 | 3 | LG-0329-4 | 0.5 |
| 49 | 5 | 3 | LG-0329-4 | 0.5 |
| 53 | 5 | 3 | LG-0329-4 | 0.5 |
| 0 | 10 | 1 | LG-0334-1 | 1 |
| 17 | 8 | 1 | LG-0334-1 | 0.8 |
| 20 | 4 | 1 | LG-0334-1 | 0.4 |
| 24 | 4 | 1 | LG-0334-1 | 0.4 |
| 41 | 4 | 1 | LG-0334-1 | 0.4 |
| 44 | 3 | 1 | LG-0334-1 | 0.3 |
| 49 | 3 | 1 | LG-0334-1 | 0.3 |
| 0 | 10 | 2 | LG-0334-1 | 1 |
| 17 | 1 | 2 | LG-0334-1 | 0.1 |
| 20 | 1 | 2 | LG-0334-1 | 0.1 |
| 24 | 1 | 2 | LG-0334-1 | 0.1 |
| 41 | 1 | 2 | LG-0334-1 | 0.1 |
| 44 | 1 | 2 | LG-0334-1 | 0.1 |
| 48 | 1 | 2 | LG-0334-1 | 0.1 |
| 0 | 10 | 3 | LG-0334-1 | 1 |
| 5 | 10 | 3 | LG-0334-1 | 1 |
| 21 | 5 | 3 | LG-0334-1 | 0.5 |
| 25 | 5 | 3 | LG-0334-1 | 0.5 |
| 29 | 5 | 3 | LG-0334-1 | 0.5 |
| 45 | 5 | 3 | LG-0334-1 | 0.5 |
| 49 | 5 | 3 | LG-0334-1 | 0.5 |
| 53 | 5 | 3 | LG-0334-1 | 0.5 |
| 0 | 10 | 1 | LG-0334-2 | 1 |
| 17 | 6 | 1 | LG-0334-2 | 0.6 |
| 20 | 3 | 1 | LG-0334-2 | 0.3 |
| 24 | 3 | 1 | LG-0334-2 | 0.3 |
| 41 | 3 | 1 | LG-0334-2 | 0.3 |
| 44 | 3 | 1 | LG-0334-2 | 0.3 |
| 49 | 3 | 1 | LG-0334-2 | 0.3 |
| 0 | 10 | 2 | LG-0334-2 | 1 |
| 17 | 0 | 2 | LG-0334-2 | 0 |
| 20 | 0 | 2 | LG-0334-2 | 0 |
| 24 | 0 | 2 | LG-0334-2 | 0 |
| 41 | 0 | 2 | LG-0334-2 | 0 |
| 44 | 0 | 2 | LG-0334-2 | 0 |
| 48 | 0 | 2 | LG-0334-2 | 0 |
| 0 | 10 | 3 | LG-0334-2 | 1 |
| 5 | 10 | 3 | LG-0334-2 | 1 |
| 21 | 5 | 3 | LG-0334-2 | 0.5 |
| 25 | 4 | 3 | LG-0334-2 | 0.4 |
| 29 | 4 | 3 | LG-0334-2 | 0.4 |
| 45 | 4 | 3 | LG-0334-2 | 0.4 |
| 49 | 4 | 3 | LG-0334-2 | 0.4 |
| 53 | 4 | 3 | LG-0334-2 | 0.4 |
| 0 | 10 | 1 | LG-0335-1 | 1 |
| 17 | 8 | 1 | LG-0335-1 | 0.8 |
| 20 | 3 | 1 | LG-0335-1 | 0.3 |
| 24 | 3 | 1 | LG-0335-1 | 0.3 |
| 41 | 2 | 1 | LG-0335-1 | 0.2 |
| 44 | 2 | 1 | LG-0335-1 | 0.2 |
| 49 | 2 | 1 | LG-0335-1 | 0.2 |
| 0 | 10 | 2 | LG-0335-1 | 1 |
| 17 | 2 | 2 | LG-0335-1 | 0.2 |
| 20 | 2 | 2 | LG-0335-1 | 0.2 |
| 24 | 2 | 2 | LG-0335-1 | 0.2 |
| 41 | 2 | 2 | LG-0335-1 | 0.2 |
| 44 | 2 | 2 | LG-0335-1 | 0.2 |
| 48 | 2 | 2 | LG-0335-1 | 0.2 |
| 0 | 10 | 3 | LG-0335-1 | 1 |
| 5 | 10 | 3 | LG-0335-1 | 1 |
| 21 | 7 | 3 | LG-0335-1 | 0.7 |
| 25 | 6 | 3 | LG-0335-1 | 0.6 |
| 29 | 6 | 3 | LG-0335-1 | 0.6 |
| 45 | 6 | 3 | LG-0335-1 | 0.6 |
| 49 | 6 | 3 | LG-0335-1 | 0.6 |
| 53 | 6 | 3 | LG-0335-1 | 0.6 |
| 0 | 10 | 1 | PA14 | 1 |
| 17 | 7 | 1 | PA14 | 0.7 |
| 20 | 1 | 1 | PA14 | 0.1 |
| 24 | 1 | 1 | PA14 | 0.1 |
| 41 | 1 | 1 | PA14 | 0.1 |
| 44 | 1 | 1 | PA14 | 0.1 |
| 49 | 1 | 1 | PA14 | 0.1 |
| 0 | 10 | 2 | PA14 | 1 |
| 17 | 6 | 2 | PA14 | 0.6 |
| 20 | 4 | 2 | PA14 | 0.4 |
| 24 | 2 | 2 | PA14 | 0.2 |
| 41 | 1 | 2 | PA14 | 0.1 |
| 44 | 1 | 2 | PA14 | 0.1 |
| 48 | 1 | 2 | PA14 | 0.1 |
| 0 | 10 | 3 | PA14 | 1 |
| 5 | 10 | 3 | PA14 | 1 |
| 21 | 2 | 3 | PA14 | 0.2 |
| 25 | 1 | 3 | PA14 | 0.1 |
| 29 | 1 | 3 | PA14 | 0.1 |
| 45 | 1 | 3 | PA14 | 0.1 |
| 49 | 0 | 3 | PA14 | 0 |
| 53 | 0 | 3 | PA14 | 0 |
| 0 | 10 | 1 | PAO1 | 1 |
| 17 | 3 | 1 | PAO1 | 0.3 |
| 20 | 2 | 1 | PAO1 | 0.2 |
| 24 | 2 | 1 | PAO1 | 0.2 |
| 41 | 2 | 1 | PAO1 | 0.2 |
| 44 | 2 | 1 | PAO1 | 0.2 |
| 49 | 2 | 1 | PAO1 | 0.2 |
| 0 | 10 | 2 | PAO1 | 1 |
| 17 | 6 | 2 | PAO1 | 0.6 |
| 20 | 5 | 2 | PAO1 | 0.5 |
| 24 | 5 | 2 | PAO1 | 0.5 |
| 41 | 5 | 2 | PAO1 | 0.5 |
| 44 | 5 | 2 | PAO1 | 0.5 |
| 48 | 5 | 2 | PAO1 | 0.5 |
| 0 | 10 | 3 | PAO1 | 1 |
| 5 | 10 | 3 | PAO1 | 1 |
| 21 | 7 | 3 | PAO1 | 0.7 |
| 25 | 6 | 3 | PAO1 | 0.6 |
| 29 | 6 | 3 | PAO1 | 0.6 |
| 45 | 6 | 3 | PAO1 | 0.6 |
| 49 | 6 | 3 | PAO1 | 0.6 |
| 53 | 6 | 3 | PAO1 | 0.6 |
| 0 | 10 | 1 | PBS | 1 |
| 17 | 9 | 1 | PBS | 0.9 |
| 20 | 9 | 1 | PBS | 0.9 |
| 24 | 8 | 1 | PBS | 0.8 |
| 41 | 8 | 1 | PBS | 0.8 |
| 44 | 8 | 1 | PBS | 0.8 |
| 49 | 8 | 1 | PBS | 0.8 |
| 0 | 10 | 2 | PBS | 1 |
| 17 | 9 | 2 | PBS | 0.9 |
| 20 | 9 | 2 | PBS | 0.9 |
| 24 | 9 | 2 | PBS | 0.9 |
| 41 | 9 | 2 | PBS | 0.9 |
| 44 | 9 | 2 | PBS | 0.9 |
| 48 | 9 | 2 | PBS | 0.9 |
| 0 | 10 | 3 | PBS | 1 |
| 5 | 10 | 3 | PBS | 1 |
| 21 | 9 | 3 | PBS | 0.9 |
| 25 | 9 | 3 | PBS | 0.9 |
| 29 | 9 | 3 | PBS | 0.9 |
| 45 | 9 | 3 | PBS | 0.9 |
| 49 | 9 | 3 | PBS | 0.9 |
| 53 | 9 | 3 | PBS | 0.9 |
| 0 | 10 | 1 | SM-0161-4 | 1 |
| 17 | 8 | 1 | SM-0161-4 | 0.8 |
| 20 | 7 | 1 | SM-0161-4 | 0.7 |
| 24 | 5 | 1 | SM-0161-4 | 0.5 |
| 41 | 4 | 1 | SM-0161-4 | 0.4 |
| 44 | 4 | 1 | SM-0161-4 | 0.4 |
| 49 | 4 | 1 | SM-0161-4 | 0.4 |
| 0 | 10 | 2 | SM-0161-4 | 1 |
| 17 | 0 | 2 | SM-0161-4 | 0 |
| 20 | 0 | 2 | SM-0161-4 | 0 |
| 24 | 0 | 2 | SM-0161-4 | 0 |
| 41 | 0 | 2 | SM-0161-4 | 0 |
| 44 | 0 | 2 | SM-0161-4 | 0 |
| 48 | 0 | 2 | SM-0161-4 | 0 |
| 0 | 10 | 3 | SM-0161-4 | 1 |
| 5 | 10 | 3 | SM-0161-4 | 1 |
| 21 | 5 | 3 | SM-0161-4 | 0.5 |
| 25 | 4 | 3 | SM-0161-4 | 0.4 |
| 29 | 4 | 3 | SM-0161-4 | 0.4 |
| 45 | 4 | 3 | SM-0161-4 | 0.4 |
| 49 | 4 | 3 | SM-0161-4 | 0.4 |
| 53 | 4 | 3 | SM-0161-4 | 0.4 |
| 0 | 10 | 1 | Uninfected | 1 |
| 17 | 10 | 1 | Uninfected | 1 |
| 20 | 9 | 1 | Uninfected | 0.9 |
| 24 | 9 | 1 | Uninfected | 0.9 |
| 41 | 9 | 1 | Uninfected | 0.9 |
| 44 | 9 | 1 | Uninfected | 0.9 |
| 49 | 9 | 1 | Uninfected | 0.9 |
| 0 | 10 | 2 | Uninfected | 1 |
| 17 | 10 | 2 | Uninfected | 1 |
| 20 | 10 | 2 | Uninfected | 1 |
| 24 | 10 | 2 | Uninfected | 1 |
| 41 | 10 | 2 | Uninfected | 1 |
| 44 | 10 | 2 | Uninfected | 1 |
| 48 | 10 | 2 | Uninfected | 1 |
| 0 | 10 | 3 | Uninfected | 1 |
| 5 | 10 | 3 | Uninfected | 1 |
| 21 | 10 | 3 | Uninfected | 1 |
| 25 | 10 | 3 | Uninfected | 1 |
| 29 | 10 | 3 | Uninfected | 1 |
| 45 | 10 | 3 | Uninfected | 1 |
| 49 | 10 | 3 | Uninfected | 1 |
| 53 | 10 | 3 | Uninfected | 1 |

**
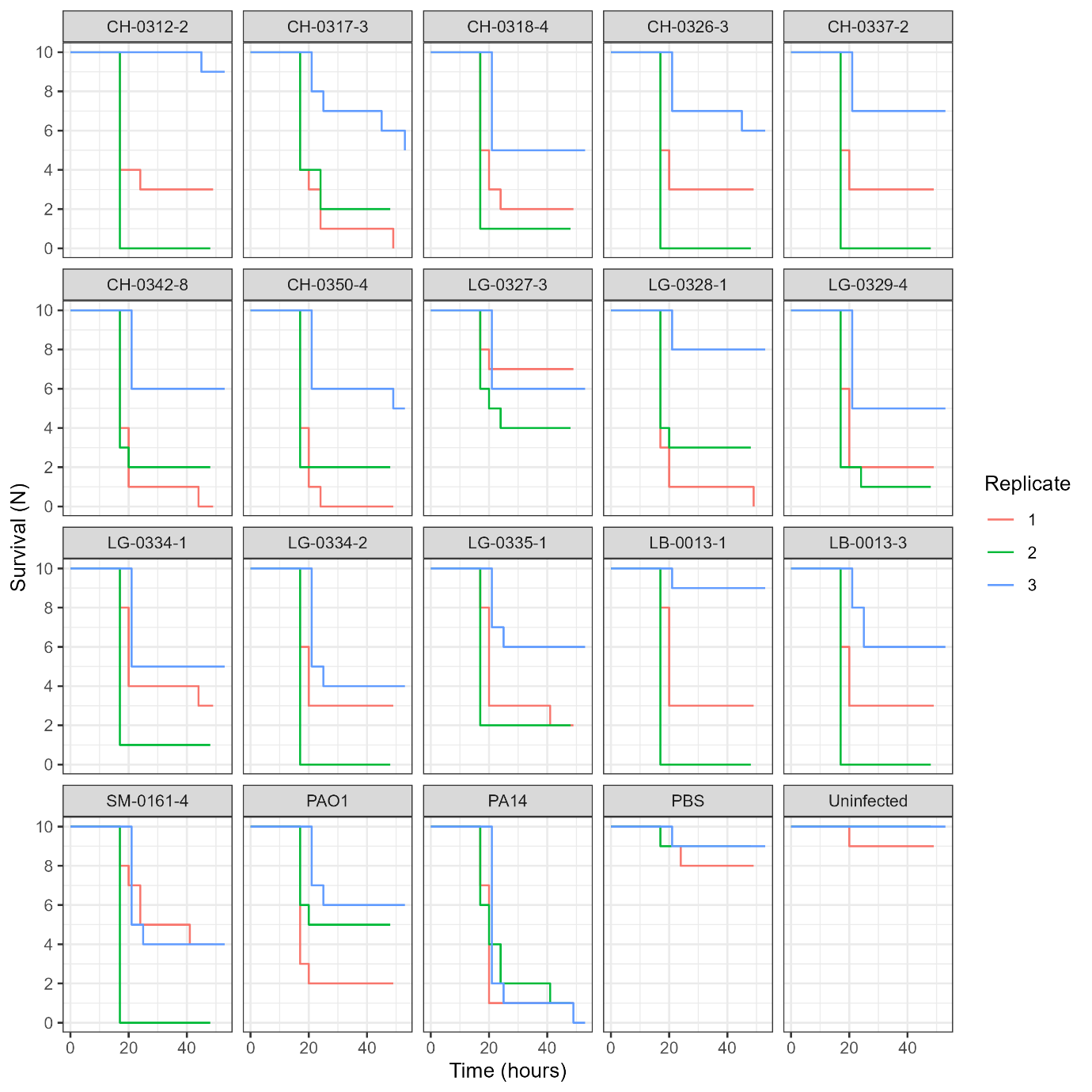
**

**Figure S6**. Survival of *Galleria mellonella* larvae over time, after being infected with different strains of *P. aeruginosa* strains collected from food, a low virulence control (PAO1), a high virulence control (PA14), PBS and uninfected, coloured by replicate.

**
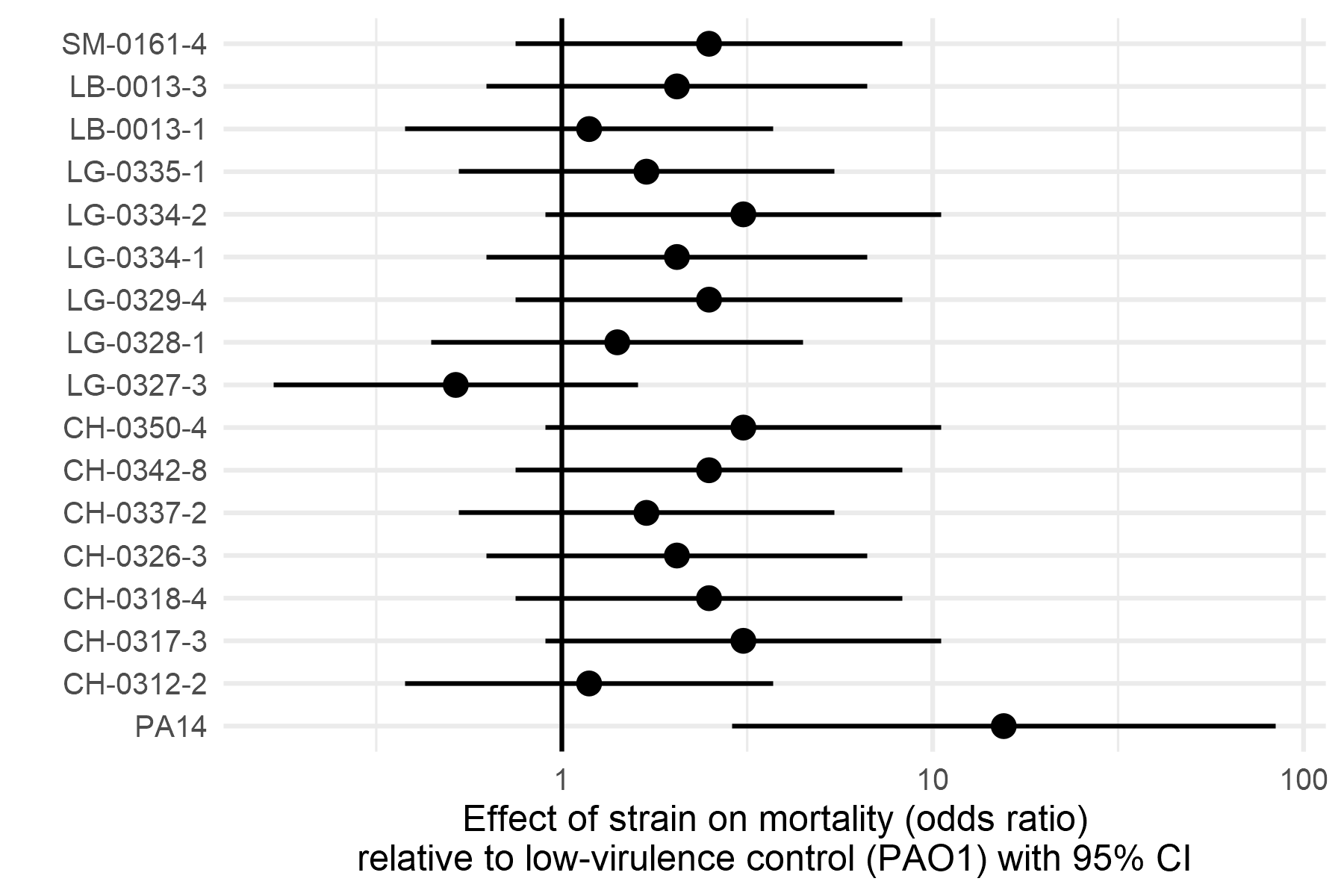
**

**Figure S7.** Odds ratio (with 95% CI) corresponding the effect of inoculation with food-recovered *P.* aeruginosa and a high virulence control (PA14) on the survival of *Galleria mellonella* larvae compared to the low virulence control (PAO1) estimated using a mixed effects logistic regression model.

**Table S8**. Mixed effects logistic regression model output of the survival of *Galleria mellonella* larvae inoculated with different *P. aeruginosa* strains compared to the low virulence control (PAO1).

| Coefficient | Odds ratio | Standard error | z-value | p-value |
| --- | --- | --- | --- | --- |
| PA14 | 15.55 | 13.39 | 3.185 | 1.450 x 10^-3^ |
| CH-0312-2 | 1.185 | 0.6918 | 0.2920 | 0.7706 |
| CH-0317-3 | 3.088 | 1.933 | 1.801 | 7.166 x 10^-2^ |
| CH-0318-4 | 2.496 | 1.529 | 1.493 | 0.1356 |
| CH-0326-3 | 2.044 | 1.232 | 1.187 | 0.2353 |
| CH-0337-2 | 1.692 | 1.006 | 0.8850 | 0.3763 |
| CH-0342-8 | 2.496 | 1.529 | 1.493 | 0.1356 |
| CH-0350-4 | 3.088 | 1.933 | 1.801 | 7.166 x 10^­-2^ |
| LG-0327-3 | 0.5181 | 0.2988 | -1.140 | 0.2542 |
| LG-0328-1 | 1.412 | 0.8307 | 0.5860 | 0.5576 |
| LG-0329-4 | 2.496 | 1.529 | 1.493 | 0.1356 |
| LG-0334-1 | 2.044 | 1.232 | 1.187 | 0.2353 |
| LG-0334-2 | 3.088 | 1.933 | 1.801 | 7.166 x 10^‑2^ |
| LG-0335-1 | 1.692 | 1.006 | 0.8850 | 0.3763 |
| LB-0013-1 | 1.185 | 0.6918 | 0.2920 | 0.7706 |
| LB-0013-3 | 2.044 | 1.232 | 1.187 | 0.2353 |
| SM-0161-4 | 2.496 | 1.529 | 1.493 | 0.1356 |

**References**

1. Cheng CM, Doyle MP, Luchansky JB. Identification of *Pseudomonas fluorescens* strains isolated from raw pork and chicken that produce siderophores antagonistic towards foodborne pathogens. J Food Prot. 1995;58:1340–4.

2. Timm CM, Campbell AG, Utturkar SM, Jun S-R, Parales RE, Tan WA, et al. Metabolic functions of *Pseudomonas fluorescens* strains from *Populus deltoides* depend on rhizosphere or endosphere isolation compartment. Front Microbiol. 2015;6:1–13.

3. Tryfinopoulou P, Tsakalidou E, Nychas GJE. Characterization of *Pseudomonas* spp. associated with spoilage of gilt-head sea bream stored under various conditions. Appl Environ Microbiol. 2002;68:65–72.

4. Ercolini D, Russo F, Blaiotta G, Pepe O, Mauriello G, Villani F. Simultaneous detection of *Pseudomonas fragi*, *P. lundensis*, and *P. putida* from meat by use of a multiplex PCR assay targeting the *carA* gene. Appl Environ Microbiol. 2007;73:2354–9.

5. Selvakumar G, Joshi P, Nazim S, Mishra PK, Bisht JK, Gupta HS. Phosphate solubilization and growth promotion by *Pseudomonas fragi* CS11RH1 (MTCC 8984), a psychrotolerant bacterium isolated from a high altitude Himalayan rhizosphere. Biologia (Bratisl). 2009;64:239–45.

6. Lee HS, Kwon M, Heo S, Kim MG, Kim G-B. Characterization of the biodiversity of the spoilage microbiota in chicken meat using next generation sequencing and culture dependent approach. Korean J food Sci Anim Resour. 2017;37:535–41.

7. Lin H, Hu S, Liu R, Chen P, Ge C, Zhu B, et al. Genome sequence of *Pseudomonas koreensis* CRS05-R5, an antagonistic bacterium isolated from rice paddy field. Front Microbiol. 2016;7:1–5.

8. Geels FP, Schippers B. Selection of antagonistic fluorescent *Pseudomonas* spp. and their root colonization and persistence following treatment of seed potatoes. J Phytopathol. 1983;108:193–206.

9. Behrendt U, Ulrich A, Schumann P. Fluorescent pseudomonads associated with the phyllosphere of grasses; *Pseudomonas trivialis* sp nov., *Pseudomonas poae* sp nov and *Pseudomonas congelans* sp nov. Int J Syst Evol Microbiol. 2003;53:1461–9.

10. Xia Q, Rufty T, Shi W. Predominant microbial colonizers in the root endosphere and rhizosphere of turfgrass systems: *Pseudomonas veronii*, *Janthinobacterium lividum*, and *Pseudogymnoascus* spp. Front Microbiol. 2021;12:1–13.

11. Balaban M, Moshiri N, Mai U, Jia X, Mirarab S. TreeCluster: Clustering biological sequences using phylogenetic trees. PLoS One. 2019;14:1–20.
